# Searching for Cancer Information on the Internet: Analyzing Natural Language Search Queries

**DOI:** 10.2196/jmir.5.4.e31

**Published:** 2003-12-11

**Authors:** Judith L Bader, Mary Frances Theofanos

**Affiliations:** ^1^National Cancer InstituteOffice of CommunicationsCancer Information Products and ServicesCommunications Technology BranchBethesda MDUSA

**Keywords:** Cancer, Internet, search engines, natural language processing

## Abstract

**Background:**

Searching for health information is one of the most-common tasks performed by Internet users. Many users begin searching on popular search engines rather than on prominent health information sites. We know that many visitors to our (National Cancer Institute) Web site, cancer.gov, arrive via links in search engine result.

**Objective:**

To learn more about the specific needs of our general-public users, we wanted to understand what lay users really wanted to know about cancer, how they phrased their questions, and how much detail they used.

**Methods:**

The National Cancer Institute partnered with AskJeeves, Inc to develop a methodology to capture, sample, and analyze 3 months of cancer-related queries on the Ask.com Web site, a prominent United States consumer search engine, which receives over 35 million queries per week. Using a benchmark set of 500 terms and word roots supplied by the National Cancer Institute, AskJeeves identified a test sample of cancer queries for 1 week in August 2001. From these 500 terms only 37 appeared ≥ 5 times/day over the trial test week in 17208 queries. Using these 37 terms, 204165 instances of cancer queries were found in the Ask.com query logs for the actual test period of June-August 2001. Of these, 7500 individual user questions were randomly selected for detailed analysis and assigned to appropriate categories. The exact language of sample queries is presented.

**Results:**

Considering multiples of the same questions, the sample of 7500 individual user queries represented 76077 queries (37% of the total 3-month pool). Overall 78.37% of sampled Cancer queries asked about 14 specific cancer types. Within each cancer type, queries were sorted into appropriate subcategories including at least the following: General Information, Symptoms, Diagnosis and Testing, Treatment, Statistics, Definition, and Cause/Risk/Link. The most-common specific cancer types mentioned in queries were Digestive/Gastrointestinal/Bowel (15.0%), Breast (11.7%), Skin (11.3%), and Genitourinary (10.5%). Additional subcategories of queries about specific cancer types varied, depending on user input. Queries that were not specific to a cancer type were also tracked and categorized.

**Conclusions:**

Natural-language searching affords users the opportunity to fully express their information needs and can aid users naïve to the content and vocabulary. The specific queries analyzed for this study reflect news and research studies reported during the study dates and would surely change with different study dates. Analyzing queries from search engines represents one way of knowing what kinds of content to provide to users of a given Web site. Users ask questions using whole sentences and keywords, often misspelling words. Providing the option for natural-language searching does not obviate the need for good information architecture, usability engineering, and user testing in order to optimize user experience.

## Introduction

For members of the general public who use the Internet, many seek medical information [1- 6]. According to a recent systematic review of 24 peer-reviewed publications describing the proportions of Internet users among various populations of cancer patients in the developed world, about 39% of cancer patients are using the Internet directly, and in addition, 15% to 20% of persons with cancer use the Internet "indirectly" through family and friends [7]. Studies have evaluated information-seeking behavior on the Internet by cancer patients generally [8- 10], their companions [11,12], and patients with the following common specific cancer diagnoses: breast [13- 16], prostate [17,18], lung [19], and gastrointestinal cancers [20]. Studies have also evaluated information gathering by cancer patients undergoing radiotherapy [21] and chemotherapy [22], and those from centers outside of North America [23,24]. Individuals from certain disadvantaged groups have been shown to seek medical information online less frequently and with more difficulty [7,25,26].

Eysenbach and Kohler found that general consumers search for medical content using search engines rather than medical portals or sites of medical societies or libraries [27]. Newly-diagnosed cancer patients and their families often start their searches as users less sophisticated in Web and medical terminology. They too commonly begin searching on popular search engines rather than on prominent cancer-information sites. We know that many visitors to our own Web site [28] arrive via search engine result links.

To better understand users' needs this research aimed to establish what lay users really want to know when they search online for cancer information. To do this we evaluated data from Ask.com [29], a popular natural-language-processing (NLP) search engine. Natural-language-processing search engines allow users to create queries using whole phrases and sentences of any length, rather than just key words.

Earlier reports of this project have been published in abstract form only. The abstracts reported a brief project summary [30], and data specific for breast cancer [31] and gastrointestinal cancer [20]. This is the first comprehensive report of the entire project.

## Methods

The National Cancer Institute (NCI) partnered with AskJeeves, Inc to develop a methodology to capture, sample, and analyze 3 months of cancer-related queries on the Ask.com Web site, a prominent US natural-language-processing consumer search engine. At the time of the project, Ask.com was receiving over 35 million queries per month.

### Search Terms

An NCI oncologist (JLB) developed a benchmark set of 500 terms and word roots that were matched against actual AskJeeves user queries. Most terms and word roots were from the NCI dictionary on the NCI Web site [32]. NCI also suggested additional terms not included in the dictionary. These terms related to anatomy, organ systems, treatments, pharmaceuticals, treatment and diagnostic procedures, genetics, epidemiology, and pathology.

**Table 1:** Top 37 search terms and roots with ≥ 5 queries per week during test week

**Term**	**Actual Queries During Test Week**	**% of Total Queries**
*cancer*	9765	56.75
*tumor*	1396	8.11
*carcino*	656	3.81
*leukemia*	635	3.69
lymphom*	419	2.43
chemotherapy	378	2.20
biopsy/biopsies	375	2.18
*melano*	348	2.02
*sarcoma*	294	1.71
*dysplasia*	255	1.48
hodgkin*	245	1.42
MRI	214	1.24
clinical trial	187	1.09
mammogr	175	1.02
maligna*	170	0.99
*metasta*	155	0.90

"*" is a placeholder for the part of the search term before or after the root.

The test sample of these 500 words and roots was used to filter cancer queries from the Ask.com Web site for 1 week in August 2001. From these 500 terms, only 37 appeared ≥ 5 times per day over the trial week. The list of 37 terms (plus common misspellings) yielded 17208 queries for the test week. The frequency of each term is shown in Table 1. Queries with common misspellings, (eg, prostate and prostrate, biopsy and biopsey, leukemia and lukemia, chemotherapy and chemothereapy) were captured and analyzed. It was felt that the cut off of 5 times per day (≥ 35 times per week) would capture the key queries and include any common query topic, since even with a frequency of 35 queries a week, the majority of these terms accounted for less than 1% of the total population. Of the original 500 terms supplied by NCI, only 7% (35/500) appeared in the logs at a high frequency, but this 7% accounts for over 37% of user queries identified as cancer related on Ask.com during the study period.

### Collecting Queries and Sampling

The process used for collecting and sampling queries is outlined in Figure 1. Using the 37 terms to search the Ask.com query logs, 204165 instances of cancer-related queries were found for June, July, and August 2001. Of these queries, 7500 individual user questions were randomly selected by AskJeeves for detailed analysis (see Appendix 1).

**Figure 1:** Processing of cancer queries on Ask.com

Very often there were multiples of the same questions. Thus, these 7500 queries actually represented 76077 queries that were entered into Ask.com, about 37% (76077/204164) of all queries identified as cancer-related from the 3 month period of log analysis. For example, a user question might be "Where can I find information about breast cancer?" This individual example represents 1 user question, but might have been queried by more than 100 people on any given day. Each query was counted only once.

### Sampling Issues

The random sample of 7500 individual queries provides a confidence interval of 1.11% at a confidence level of 95%. This means that even if more samples were taken from 204165 queries, 95% of those samples should not be off by more than 1.1%. While this means that the samples themselves would not vary more than 1.1% over 95% of the samples taken, as the data are categorized and classified, in effect smaller and smaller samples are taken. Therefore, to offset this problem additional queries were examined, even though a smaller sample would still provide a high degree of confidence in the results.

In other words, although broad generalizations—such as "breast cancer accounts for 25% of all cancer queries"—can be easily presented, a large sample size is required to break down data far enough to conclude that when users ask about breast cancer, they are most often asking about specific types of treatments.

### Highest-Level Categories for Queries

User queries were assigned to a set of 6 highest-level categories (as shown in Table 2):

- Cancer (ie, specifically mentioning a cancer type)
- General Research
- Treatment
- Diagnosis and Testing
- Cause/Risk/Link
- Coping

**Table 2:** Highest-level categories for queries

**Highest-Level Category**	**Number of Queries**	Percent of All Sampled Queries*
Cancer †	59619	78.37
General Research	7808	10.26
Treatment	3832	5.04
Diagnosis and Testing	3315	4.36
Cause/Risk/Link	1249	1.64
Coping	254	0.33
**Total**	**76077**

^*^ Percentages do not sum to 100% due to rounding.

^†^ ie, specifically mentioning a cancer type.

Highest-level categories were created in a collaborative effort between the AskJeeves data-analysis team and NCI staff before the study period began, but the final category titles were revised as the actual queries were analyzed. The initial categories were based on user queries entered into Ask.com and a variety of online sources, such as NCI's online dictionary [32] and NCI's Physician Data Query (PDQ) [33].

The highest-level categories were populated using proprietary AskJeeves filters and automated-analysis tools that sorted queries according to specific types of cancers, or—in the absence of mentioning a specific cancer type—whether the query asked about other areas such as Treatment or Coping. (AskJeeves did not share the filters and automated analysis tools with the authors.) Queries that could not be sorted by the filters and automated-analysis tools were placed in a temporarily-uncategorized category; they were categorized during the next step (reading and analysis).

Reading and analyzing each individual query not only verified the automated process, but also helped to refine existing categories and create new categories and subcategories, as appropriate. For example, without this type of analysis, the query "Where can I find a Web site with information on using high protein food to fight Breast cancer?" might have been left under Breast Cancer > Media and Organizations > Web sites (where ">" indicates a change in category level). This would not be correct, as the true user intent was to inquire about Alternative Treatments. As a result, under the category Breast Cancer > Treatment, "Alternative" was added to the Breast Cancer > Treatment category analysis as a subtopic. (Treatment—without a specific cancer site designated—is both a highest-level category and a subcategory under Breast Cancer and under most cancer types.)

Approximately 78% of all categorized queries from the sample referenced a particular type of Cancer, and were placed in the highest-level category Cancer. An example of this kind of query would be "Where can I find information about Breast Cancer?" (This query would be classified as Cancer > Breast Cancer > General Information.) Any query that did not mention a specific kind of Cancer, even though the question was about cancer, was placed on 1 of the 5 other highest-level categories. An example of this type of query would be "Where can I find information on cancer treatment with radiation?" This query was assigned to the Radiation subcategory in the highest-level category Treatment (ie, it was classified as Treatment > Radiation).

Queries that did not relate to a specific Cancer type were placed in 1 of the 5 other highest-level categories: General Research, Treatment, Diagnosis and Testing, Cause/Risk/Link, or Coping. For example the query "How does smoking cause cancer?" would be placed in the Cause/Risk/Link category, as it did not refer to any specific type of cancer.

### "Cancer" Queries (Related to Specific Cancer Types)

As shown in Table 3, there were 14 cancer types (N = 59619 queries) selected as subcategories of the Cancer highest-level category. For cancer types with the most-frequent queries, like Digestive/Gastrointestinal/Bowel (D/G/B), Breast, and Genitourinary, there were enough queries to populate standard subcategories like General Information, Treatment, Symptoms, Diagnosis and Testing, and Cause/Risk/Link. These common cancer types often warranted the creation of customized subcategories, like Breast > Media and Organizations > Web sites. For the less-common cancer type queries, like Bile (duct) in D/G/B, few queries were received and only those in General Information are shown.

**Table 3:** Cancer types

**Type within Top-Level Cancer Category**	**Number of Queries**	% Queries in Cancer Category*†	% Queries in This Report*‡
Digestive/Gastrointestinal/Bowel (D/G/B)	8959	15.0	11.8
Breast	6953	11.7	9.1
Skin	6709	11.3	8.8
Genitourinary	6250	10.5	8.2
Hematologic/Blood	5448	9.2	7.2
Gynecological	5344	9.0	7.0
Lung	4630	7.8	6.1
Soft Tissue/Muscle	3954	6.6	5.2
Lymphoma	3333	5.6	4.4
Head and Neck	2522	4.2	3.3
Brain/Neurological	1852	3.1	2.4
Miscellaneous Cancer	1633	2.7	2.1
Bone	1429	2.4	1.9
Pediatric	603	1.0	0.8

^*^ Percentages do not sum to 100% due to rounding.

^†^ Denominator (N = 59619) was the total number of queries about specific types in the Cancer category.

^‡^ Denominator (N = 76077) was the total number of queries analyzed in this report.

### Privacy Issues

Although NCI helped create the search terms and the categories into which the analyzed data was placed, NCI did not have access to: the raw query logs at AskJeeves, any information about what AskJeeves users did with the searches generated on the AskJeeves Web site (ie, what links they picked), or the identities of any users of the Ask.com Web site. NCI did not require permission from the Institutional Review Board.

## Results

### Frequency of Top-Level Categories

As shown in Table 2, The 6 highest-level categories in order of decreasing frequency of queries were:

- Cancer (N = 59619, 78.37%)
- General Research (N = 7808, 10.26%)
- Treatment (N = 3832, 5.04%)
- Diagnosis and Testing (N = 3315, 4.36%)
- Cause/Risk/Link (N = 1249, 1.64%)
- Coping (N = 254, 0.33%)

The data in Table 2 indicate that the great majority of users asked for information about specific types of cancers, but rarely asked about a Treatment option or Diagnosis and Testing procedure without specifying the particular cancer about which they were concerned. Similarly, users asked few queries about general Symptoms of cancer unrelated to a specific type of cancer (see Diagnosis and Testing > Symptoms, N = 473, 14.27%). An example would be "what are some symptoms of cancer?"

### Subdividing Cancer Queries

Table 3 breaks down the highest-level category Cancer queries (N= 59619) into more specific cancer types. In order of decreasing frequency within the Cancer category, the 14 subcategories were:

- Digestive/Gastrointestinal/Bowel (D/G/B) (N = 8959, 15.0%)
- Breast (N = 6953, 11.7%)
- Skin (N = 6709, 11.3%)
- Genitourinary (N = 6250, 10.5%)
- Hematologic/Blood (N = 5448, 9.2%)
- Gynecological (N = 5344, 9.0%)
- Lung (N = 4630, 7.8%)
- Soft Tissue/Muscle (N = 3954, 6.6%)
- Lymphoma (N = 3333, 5.6%)
- Head and Neck (N = 2522, 4.2%)
- Brain and Neurological (N = 1852, 3.1%)
- Miscellaneous (N = 1633, 2.7%)
- Bone (N = 1429, 2.4%)
- Pediatric (N = 603, 1.0%)

Any query specifically mentioning a cancer type by name, was assigned to that subcategory. For example, questions about Breast-Cancer-specific Treatment, Diagnosis and Testing, Causes, and Coping are found in the Cancer > Breast Cancer category, within 1 of the 10 subcategories displaying Breast Cancer information. All questions about Leukemia or Myeloma would be found in Hematologic/Blood, Hodgkin's Disease queries in Lymphoma, and Esophageal cancer questions in D/G/B.

The number of subcategories assigned to each of the 14 different cancer types varied somewhat and was driven by the nature and number of the specific queries in those cancer types.

### Detailed Analysis of Queries

The detailed categorizations and verbatim display of examples of sampled queries are shown in Appendix 1. There is a breakdown of all the 14 cancer types within the highest-level category Cancer as well as a breakdown of queries within the 5 other highest-level categories not referencing any particular cancer type. These 19 are arranged alphabetically in the Appendix.

Major observations about the 19 categories and subcategories are noted below, in the order they appear in the Appendix. Our comments emphasize issues related to requested cancer content more than technology issues related to the natural language processing.

#### 1.0 Bone Cancer

As shown in Appendix 1, there were 1429 queries about Bone Cancer. The vast majority of Bone Cancer queries asked for General Information (N = 1107, 78%). An example of this category would be: "Where is information on bone cancer?" Users asked questions about Bone Cancers linked to various sites of Anatomy as well as certain Histologies. There were some questions related to Bone Cancers in teenagers that were assigned to this category, rather than the Pediatric category. There were more questions about Diagnosis and Testing (N = 64, 4.48%) and Symptoms (N = 135, 9.45%) than Treatment (N = 26, 1.82%).

#### 2.0 Brain and Neurological Cancer

Of the 1852 Brain and Neurological Cancers queries, General Information accounted for the vast majority (N = 1323, 71.44%). There were 427 (23.1%) questions about specific cancer types in this category. Some cancer types queries asked about Medulloblastoma, which is typically but not always a Pediatric tumor. As with Bone Cancer above, some questions could have been meaningfully assigned to more than 1 top-level Cancer site category. In this category there were more queries about Symptoms (N = 259, 13.98%) than Treatment (N = 112, 6.05%).

#### 3.0 Breast Cancer

As shown in Appendix 1, Breast Cancer was one of the simpler cancer types, from a data-display standpoint. There was only 1 anatomic-cancer type and all of the individual queries for that cancer type were assigned into 1 of 10 subcategories.

The 10 top-level Breast Cancer subcategories were:

- General Information (N = 3423, 49.23%)
- Symptoms (N = 889, 12.79%)
- Treatment (N = 570, 8.20%)
- Media/Organization (N = 428, 6.16%)
- Cause/Risk/Link (N = 393, 5.65%)
- Diagnosis and Testing (N = 376, 5.41%)
- Statistics (N = 274, 3.94%)
- Pictures (N = 225, 3.24%)
- Type (N = 217, 3.12%)
- Definition (N = 158, 2.27%)

Nine of the 10 Breast Cancer subcategories were analyzed in detail in Appendix 1. The tenth, Pictures, did not require further analysis. Most queries asked for General Information.

There were more frequent queries about Breast Cancer (N = 6953) than any other cancer type. This may not be apparent from Table 3, which appears to show more D/G/B cancers (N = 8959). However, D/G/B overall is actually composed of 10 cancer types. The most frequently queried cancer type in D/G/B was Colorectal (N = 4,801) which had fewer queries than Breast.

Even though other cancer types may have been assigned more subcategories than the 10 for Breast, the detail and the medical specificity and technical vocabulary of Breast queries appear to be the most complex than other Cancer sites, probably reflecting the sophistication of basic research and clinical data on this topic and the relative sophistication of the breast cancer information seekers.

#### 4.0 Cause and Risk

There were 1249 queries in this highest-level category. Without mentioning a specific cancer by name, there were N = 1115 (89.27%) queries about Causes and Links but only N = 134 (10.73%) about Prevention. Among the 1115 queries in the Causes and Links subcategory, the following topics were noted:

- Drugs (N = 287, 25.74%)
- Unspecified (N = 247, 22.15%) (eg, "What is cause a cancer?" [sic])
- Radiation (N = 247, 22.15%)
- Personal (N = 116, 10.40) (eg, "Can anti-persperant [sic] deodorant cause cancer?")
- Chemical/Plastics (N = 74, 6.64%)
- Environmental (N = 70, 6.28%)
- Food Supplement (N = 64, 5.74%)
- Genetic Mutation/Virus (N = 10, 0.90%)

Smoking was not in this list, probably because most queries about smoking were included under a query about a specific type of cancer, like Lung or Head and Neck.

#### 5.0 Coping

There were only 254 queries about Coping. The queries referenced Support Groups (N = 127, 50%), Pain (N= 98, 38.58%), and Depression (N = 29, 11.42%). Even though there were few questions in this highest-level category, the issue was of specific interest to NCI, which asked for this category to be created and analyzed separately.

#### 6.0 Diagnosis and Testing

There were 3315 queries in this highest-level category, which did not mention a specific cancer by name. Most were queries about specific Testing (N = 2842, 85.73%). The others (N = 473, 14.27%) were queries about Symptoms. Among Testing queries, CAT/CT scan (Computerized Axial Tomography/Computed Tomography scan) (N = 1509, 53.10%) and MRI (N = 587, 20.65%) were the most-common Testing topics, followed by Biopsy (N = 502, 17.66%).

#### 7.0 Digestive/Gastrointestinal/Bowel (D/G/B)

The presentation of data queries for D/G/B in Appendix 1 is complex because, there were 7 top-level subcategories, including General Information and 10 cancer types identified in the General Information subcategory

As shown in Appendix 1, 8959 queries for D/G/B sites were broken down into 7 subcategories:

- General Information (N = 5568, 62.15%)
- Symptoms (N = 1506, 16.81%)
- Diagnosis and Testing (N = 1125, 12.56%)
- Treatment (N = 294, 3.28%)
- Statistics (N = 184, 2.05%)
- Definition (N = 163, 1.82%)
- Cause/Risk/Link (N = 119, 1.33%)

Most queries asked for General Information. Examples of General Information queries would be "Where can I learn about the cancer esophageal cancer?"? and "Where can I find information on Stomach cancer"?

A breakdown of all D/G/B queries by cancer type is shown in the list below. The absolute numbers and percentages (of all D/G/B queries) in the list below differ from the pie diagram in Appendix 1 because the list below includes organ-type queries from General Information plus the 6 other subcategories in D/G/B.

- Colorectal (N = 4801, 53.59)
- Liver (N = 1413, 15.77%)
- Gastrointestinal (stomach) (N = 1094, 12.21%)
- Pancreas (N = 965, 10.77%)
- Bowel (N = 273, 3.05%)
- Esophagus (N = 260, 2.90%)
- Other (N = 153, 1.7%)

The organ subsites in Other include Gall Bladder, Bile Duct, Anal, and Abdominal.

As noted in Appendix 1, for D/G/B there were far more questions about Symptoms (N = 1506, 16.81%) than Treatment (N = 294, 3.28%) possibly reflecting the fact that (1) users of Ask.com were just beginning their D/G/B information seeking and (2) there is less complexity in the published Treatment data for D/G/B compared to some other cancer types, like Breast Cancer.

The terms Bowel, Gastrointestinal, Stomach, and Abdominal may have been used interchangeably by users. They appear not to recognize that queries for sigmoid, rectum, cecum, appendix, transverse colon, small bowel, and stomach (gastric) cancer would provide much more useful information.

For D/G/B, some queries about Liver Metastases were included with queries about primary Liver Cancers.

#### 8.0 General Research

There were 7808 queries assigned to the highest-level category General Research, a topic not linked to a specific cancer type. In this category the 5 most-common subcategories were:

- Research (N = 2819, 36.10%)
- Organization (N = 1656, 21.21%)
- Clinical Trials (N = 1272, 16.29%)
- Concerns (N = 1201, 15.38%)
- Pictures (N = 559, 7.16%)

Among the queries about Organization, there were 1065 queries about the American Cancer Society (ACS) and 223 about the National Cancer Institute (NCI).

Among the 1272 queries about Clinical Trials, the most-common 3 questions/topics were:

- What are ... (N = 634, 49.84%) eg, "What are clinical trials?"
- Latest ... (N = 260, 20.44%) eg, "latest cancer clinical trial research"
- Types of ... (N = 111, 8.73%) eg, "types of cancer trials"

#### 9.0 Genitourinary Cancers

In decreasing order, the frequency of Genitourinary organ-type queries (N = 6250) in all 12 Genitourinary subcategories including General Information was:

- Prostate (N = 3141, 50.26%)
- Testicular (N = 1772, 28.35%)
- Bladder (N = 708, 11.33%)
- Kidney (N = 496, 7.94%)
- Other (N = 133, 2.12%)

Although it has been estimated that there were 198100 new cases of Prostate Cancer diagnosed in the US in 2001 and only 7200 cases of Testis Cancer [34], the relative frequency of Testis Cancer queries was quite high. One possible reason might be that males diagnosed with Testis Cancer are generally much younger than those diagnosed with Prostate Cancer, and those younger individuals might be more-frequent information seekers on the Internet. It may also reflect the fact that the 2001 Tour de France bicycle race won by Lance Armstrong, a Testis Cancer survivor, was held during July, coinciding with the study period for this project.

As with most sites, the most-common Prostate Cancer questions were General Information (N = 1715, 54.6%). For Prostate Cancer, there were more questions about Treatment (N = 460, 14.65%) than Symptoms (N = 364, 11.59%). This may reflect major medical controversies about treatment options and the typically asymptomatic presentation of the disease.

For the Genitourinary category as a whole, there were more questions about Symptoms (N = 854, 13.66%) than Treatment (N = 604, 9.66%).

Expected misspellings of prostate (prostrate) were noted.

#### 10.0 Gynecological Cancers

There were 5344 queries overall. The breakdown of subcategories in decreasing frequency was:

- General Information (N = 3409, 63.79%)
- Symptoms (N = 939, 17.57%)
- Diagnosis and Testing (N = 452, 8.46%)
- Treatment (N = 247, 4.62%)
- Definition (N = 158, 2.96%)
- Cause/Risk (N = 83, 1.55%)
- Statistics (N = 42, 0.79%)
- Prevention (N = 14, 0.26%)

In decreasing order of frequency, the cancer types queried in all 8 Gynecological subcategories included the following:

- Ovarian (N = 2031, 38.00%)
- Cervical (N = 1924, 36.00%)
- Uterine (N = 606, 11.34%)
- Endometrial (N = 225, 4.21%)
- Vulvar (N = 166, 3.11%)
- Vaginal (N = 219, 4.09%)
- Other or not specified (N = 173, 3.24%)

There were nearly as many questions about Cervical Cancer as Ovarian Cancer despite the fact that in the United States in 2001 the estimated incidence of new Ovarian Cancers was about twice that of invasive Cervical Cancer [34].

There were questions about Endometrial cancer as well as Uterine cancer. These data suggest that Web site information needs to be provided using both labels.

#### 11.0 Head and Neck

There were 2522 queries overall. Most queries asked for General Information (N = 1485, 58.88%). The vocabulary used to ask about specific cancer types within General Information was:

- Throat
- Mouth
- Oral
- Tongue
- Head
- Neck

The vocabulary confirms the need to offer health information with words that are not technical like larynx, glottis, pharynx, or nasopharynx. There were 59 questions asking about Definitions of Head and Neck cancer. Specifics about cancer anatomy of this cancer type may be less familiar to the general public than other sites.

There were 422 queries asking for Pictures of Head and Neck Cancer. There were only 47 questions (1.86%) asking about Cause/Risk/Link issues, despite the fact that there is a great deal known about the Causes and Prevention of Head and Neck Cancer. There were 418 questions (16.57%) about Symptoms and but only 52 (2.06%) about Treatment.

#### 12.0 Hematologic and Blood Cancers

Among 5448 queries in this category, the 5 most common of the 12 subcategories were: General Information (N = 3781, 69.40%), Definition (N = 701, 12.96%), Symptoms (N = 539, 9.89%), Treatments (N = 175, 3.21%), and Organizations (N = 102, 187%). Within General Information users asked about Leukemia (N = 2895, 76.57%), Myeloma (N = 592, 15.66%), Bone Marrow (N = 148, 3.91%), and Blood Cancers (N = 146, 3.86%). Various misspellings of Leukemia were noted and nontechnical terms such as Blood Cancer and Bone Marrow Cancer were frequent.

#### 13.0 Lung Cancer

Lung Cancer (N = 4630) accounted for 8% of organ-type specific queries within the highest-level Cancer category. This is a disproportionately-low percentage given the relative incidence of Lung Cancer in the United States in 2001 [32]. There were more queries about Gynecological and Hematologic/Blood cancers, even though the US incidence for these is far lower.

Among Lung Cancer queries, the queries were classified as follows:

- General Information (N = 3223, 69.61%)
- Symptoms (N = 530, 11.45%)
- Cause/Risk/Link (N = 305, 6.59%)
- Treatment (N = 219, 4.73%)
- Definition (N = 150, 3.24%)
- Statistics (N = 113, 2.44%)
- Diagnosis and Testing (N = 90, 1.94%)

In the Cause/Risk/Link category of Lung Cancer, there were only N = 180 queries (59.02%) that asked generally about Causes of Lung Cancer and N = 102 queries (33.44%) that asked specifically about Smoking. There were N = 23 queries (7.54%) asking if Marijuana caused Lung Cancer.

Only N = 255 (7.91%) queries within General Information asked about Lung Cancer by (histologic cell) Type, despite the fact that this is a major determinant of triage for treatment.

For Lung Cancer > Treatment, there were 219 queries (4.73%). Most Treatment queries were Unspecified (N = 118, 53.88%), eg, "What are treatments for lung cancer?" There were 26 Treatment questions about Cure (11.87%). There were few specific questions about Medications (chemotherapy) (N = 21, 9.59%), Radiation (N = 19, 8.68%), or Surgery (N = 10, 4.57%). Although all numbers were small, there were more questions about Alternative Treatment (N = 13, 5.94%) than Surgery (N = 10, 4.57%). There were only 4 Treatment questions (1.83%) about palliative care, despite the grave prognosis for most Lung Cancers. Clearly the questions about Lung Cancer, the most-common lethal cancer, were far less sophisticated than the questions about either Breast Cancer or Prostate Cancer.

#### 14.0 Lymphomas

Among the 3333 queries about Lymphoma (including both Hodgkin's Disease and Non-Hodgkin's Lymphoma), General Information (N = 2391, 71.74%) questions were the most common. Unlike many cancer types, there was frequent mention of histologic types, as is appropriate, given the wide variety of clinically-different prognoses and treatments in this subcategory. There were many different spellings of Hodgkin's Disease.

#### 15.0 Miscellaneous Cancers

There were 1633 queries assigned to this Cancer subcategory. The Miscellaneous Cancers were:

- Endocrine (N = 901, 55.17%)
- Neoplasm (N = 272, 16.66%)
- Kaposis (N = 262, 16.04%)
- Ocular (N = 179, 10.96%)
- Germ Cell (N = 19, 1.16%)

Several of the Ocular queries, eg, Ocular Melanoma and Retinoblastoma, could have been considered for other subcategories, such as Skin and Pediatric respectively. Germ cell tumors could also have been placed in either Genitourinary or Gynecological subcategories. These ambiguities illustrate the difficulty in categorizing precise user information needs despite the use of natural language processing.

#### 16.0 Pediatric

There were only 603 Pediatric queries, and most asked about a specific cancer type (N= 403, 66.83%). There were relatively few General Information queries (N = 81, 13.43%) eg, "where can I find information on children's cancers?" Since patients with Pediatric cancers in the US are usually managed generally by pediatric oncology specialists at major regional medical centers, those seeking Pediatric cancer information are probably directed to specialized Web sites rather than general sites like Ask.com.

Of 403 queries for cancer types, the most common were Hematologic/Blood (N = 137, 34%), Neuroblastoma (N = 133, 33%), and Rhabdomyosarcoma (N = 68, 16.87%). There were only 4 questions referring to pediatric Brain and Neurological cancers. Since this is such a common Pediatric tumor type, it is possible that some Pediatric neurological tumor questions were assigned to the Brain and Neurologic Cancer category even though the questions were really meant to target a Pediatric issue.

#### 17.0 Skin Cancers

Among 6709 queries in this Cancer subcategory, 3596 (53.60%) asked for General Information. Like Lymphoma, there was frequent mention of specific Skin Cancer types (N = 2157, 32.15%), probably because of the significantly-different clinical prognoses and treatments.

Only 169 queries (2.52%) asked about Cause/Risk/Link, and 60 queries (0.89%) asked about Prevention despite the fact that so much is known about these topics and Skin Cancer.

Among Skin Cancers queried by histologic cancer type (N = 2157, 32.15%), Melanoma was the most common (N = 1707, 79.14%), even though it is far-less common than Basal Cell Skin Cancers (N = 322, 14.93%) [10]. Frequent mention of Melanoma probably reflects its more-serious prognosis and more-complicated clinical triage.

#### 18.0 Soft Tissue Cancers

There were 3954 queries in this Cancer subcategory. Although most appropriately refer to sarcomas of various types, there was a minority of misplaced queries. Some queries appear to reference conditions that are probably benign (Ganglion, Fibroid, Dysplasia, and Lipoma) and others should have been placed in different Cancer subcategories eg, Brain and Neurological (Oligodendroglioma and Glioma) These will be corrected on later analyses.

#### 19.0 Treatment

In the 3832 highest-level category queries about Treatment, most questions were about a specific Treatment Type (N = 3223. 84.11%), even though no specific cancer was mentioned. Within Treatment > Treatment Type there were many general queries about Chemotherapy (N = 2275, 70.59%). There were questions about general Radiation Therapy (N = 534, 16.57%), and few about specialized Radiation Therapy treatments like Gamma Knife, Laser, and Protons. There were more general questions about Alternative Therapies (N = 239, 7.42%) than Surgery (N = 127, 3.94%) Many Alternative Therapy questions also appear in specific organ-type subcategories, particularly Breast.

### Query Frequency Relative to US Incidence of Cancer Types

Table 4 compares the incidence of selected cancers in the United States (US) in the year 2001 with the frequency of selected site-specific cancer queries in this report. It has been estimated that there were 1268000 new cancer cases in the US in 2001 [34]. The sites in Table 4 were selected specifically because they were easiest to compare directly.

The relative percentage of specific organ-type queries exceeds the percentage of annual incidence only for rarer cancers. The difficulty of finding useful information on prominent cancer portals or with standard search engines may be one explanation, although there are others. The comparison is not meant to be definitive as there are clearly issues with validity of this comparison:

- Cancer prevalencemight be a better benchmark than incidence
- US incidence data exclude cases of in situ breast and cervix cancers as well as the very-common basal cell and squamous cell skin cancers
- Queries could have come from anywhere in the world, not just the United States
- Query total may include those who accessed the site more than once
- Queries could have come from individuals who are not newly-diagnosed patients

**Table 4:** Comparing relative annual US incidence of selected cancers and query frequency

**Cancer Site**	Estimated Number of New US Cancers Diagnosed in 2001*	% of Estimated New US Cancers in 2001*†	**Number of Cancer Site-Specific Queries in This Report**	**% Queries in Cancer Category**†‡
Digestive (D/G/B)	235700	18.6	8959	15.0
Prostate	198000	15.6	3141	5.3
Breast	193700	15.3	6953	11.7
Lung	169000	13.3	4630	7.8
Lymphoma	63600	5.0	3333	5.6
Bladder	54300	4.3	708	1.2
Uterus/Endometrial	38300	3.0	931	1.6
Head and Neck	30100	2.3	2522	4.2
Ovary	23400	1.9	2031	3.4
Brain and Neurological	17200	1.4	1852	3.1
Cervix	12900	1.0	1924	3.2
Soft Tissue	8 700	0.69	3954	6.6
Testis	7200	0.57	1772	3.0

^*^ Data from 2001 Estimated Annual US Cancer Incidence Figures (N = 1268000) [10].

^†^ Percentages in columns 3 and 5 do not add up to 100% because only selected cancers were included in this chart.

^‡^ Only selected Cancers were included in this chart. Denominator (N = 59619) was the total number of queries about specific subsites in the Cancer category.

### Other Observations

The query analysis reveals that online users generally seek information about Symptoms and Treatment for specific cancers, rather than about cancers generally. In addition, Symptom queries showed a frequency between 2 and 5 times that of Treatment queries, for most cancers.

For this study we did not specifically target queries about Adult Immune Deficiency Syndrome (AIDS), even though AIDS can often be associated with Cancer. There were 262 questions about Kaposi's Sarcoma in the Miscellaneous Cancers category.

## Discussion

General Information was the largest category for almost all cancers, probably reflecting the nature of the Ask.com consumer search engine. It is a consumer-oriented Web-wide search engine where users tend to seek general information that can help them learn either how or where they should further pursue their inquiries. It is likely the users are just starting their Web searches on Ask.com and they are not yet interested in or they do not yet know enough information to ask more-sophisticated questions. This behavior may not reflect that of users who go directly to a known cancer-information portal with a predetermined need for detailed information.

We attempted to capture and analyze all cancer-related queries, including those with correct and incorrect spellings. Misspellings were noted relatively frequently, but we have no data on the number of misspellings, as we did not target this in advance as an endpoint, and we did not have direct access to the raw data logs. Appendix 1 shows verbatim queries with examples of the misspellings. Automating help for users who enter misspelled words is a major issue for search engines in order to optimize query results. Other researchers have noted the search difficulties related to spelling of cancer search terms correctly [35].

Ask.com users entered both keyword searches and sentence-style queries, despite the fact that this is a natural-language-processing search engine. We recognize that even if users typed in a long query it was still sometimes difficult to discern absolutely what specific information the user needed, particularly since we did not have access to the links users picked.

The vocabulary employed by users of Ask.com ranged from unsophisticated to very sophisticated. This suggests that allowing users to employ less-technical language on cancer Web sites would significantly help them find the information they seek.

The queries captured for this study undoubtedly reflect the news and research studies in the public arena during the time period from June to August 2001. A different time period would certainly reflect a different distribution. Examples of the kinds of events that could affect the results include the diagnosis or death of a celebrity with cancer, the publication of a major trial about bone marrow transplantation for breast cancer, or the Food and Drug Administration approval of an important new drug.

The presence of a search engine with natural language processing on a Web site, while potentially valuable to users, does not obviate the need for good user-centered Web site design and information architecture [36]. It has been shown that searching via search engine can be minimized and user satisfaction maximized if information architecture and link titles follow appropriate guidelines [37]. Nevertheless, for less-sophisticated users, a natural-language-processing search engine can be helpful in finding the information users seek and provide enhanced success in searching.

An October 30, 2003 search of the PubMed Web site [38] of the National Library of Medicine [39] yielded 458 search results from a query for "Natural Language Processing." Most citations were from publications within the last 3 years, attesting to the currency of natural language processing as an important research topic cutting across a wide variety of research disciplines. Potential data-mining applications of this tool in medicine extend far beyond the use described in this paper.

Eysenbach and Kohler have recently developed a novel methodology, similar to the method used in this study, to estimate the actual volume and prevalence of health-related searches on the Web in relation to the total number of searches conducted daily on the Internet [40]. They collected queries from 2 search engines, Metacrawler (a search engine of search engines) [41] and Ask.com [29] (the same natural-language-processing search engine used for this report). These 2 search engines were selected because they allowed "peeking" at actual user search-query topics. They concluded that 4.5% of all searches on the Web might be health related. The queries were collected from Metacrawler between February 2001 and April 2002, and from Ask.com between February 2001 and April 2001. The first date range overlapped our study dates and the second occurred just before data collection for our study.

In summary, natural-language-processing tools such as the one used for this study are able to filter and subset raw query data into useful analysis categories. Retrieval and analysis of these data can be used to better understand the actual content users want and the level of understanding and sophistication they have when they come to the Web site. Using the information on a continuing basis can form the basis for updating content on Web sites based on the most-current user needs. If a natural-language search engine were offered on a health-information portal, for example, it could improve customer access to desired information, particularly for those users with less sophistication about content or language. Additional analyses of query results are planned for the future. Consideration has been given to piloting the use of natural language processing on subsites of our Web portal.

## Figures and Tables

**Figure A1:**
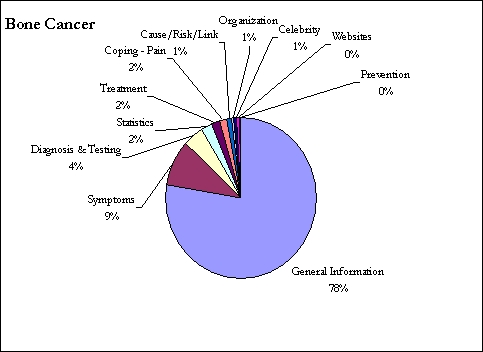
Bone Cancer

## Abbreviations

AIDS: Adult Immune Deficiency Syndrome
ACS: American Cancer Society
D/G/B: Digestive, Gastrointestinal, and Bowel
NCI: National Cancer Institute
US: United States

## Data Supplement Appendix: Data Categorization, Counts, and Charts

Click here for complete Data Supplement Appendix:

### Overview

Appendix 1 contains the counts and exact wording for all of the categorized questions from the sample of 7,500 user questions. Each category (such as *Breast or Head & Neck*) will have the highest level of breakdown on the first page, and subsequent breakdowns (if possible) on that page and following pages.

### Rounding in Pie Charts

When looking at certain pie charts in Appendix 1 there will be categories that are shown to be 0%. This is due to rounding of numbers in Microsoft Excel. The actual percentage can be seen in the tabular format.

### Additional Information in Pie Charts

The charts embedded within the Appendix can be double clicked to reveal additional information.

### Tables

Most categories are broken out into tables with 4 columns. An excerpted example is shown below before the actual data tables are displayed. It shows the breakdown of Brain and Neurological Cancer > General Information > Cancer Type. The columns contain the following information:

- The first column starts with the category name in a yellow cell. In the example shown this is Cancer Type. Below Cancer Type are the types of cancers found within that category. For other categories, these would be the representative terms for that category, ie, for a category such as Treatment there might be listings for Alternative, Chemotherapy, Surgery, and Radiation.
- The second column of the illustrative table contains the raw count of user queries for that field. As shown in the example, Astrocytoma was queried 144 times, which is 33.72% of all queries that are found in the subcategory of Cancer Type.
- The third column shows the percentages for that subcategory. These are category specific, meaning that they are percentages of only those terms within that category or subcategory. Therefore Benign cancers represent 2.81% of all Cancer Type queries and are not 2.81% of all Brain & Neurological Cancer queries. While it was not the intention to include benign queries in this analysis, a small number were captured and analyzed, and therefore appear in the tables.
- The fourth column header notes where the subcategory is in relation to the main category. In this case Cancer Type was created within the General Information category of the cancer site Brain & Neurological Cancers. The counts are also included, to illustrate that out of the total number of Brain & Neurological Cancers (N = 1852 queries), General Information queries accounted for 1323 queries, which were 72% of all Brain and Neurological Cancer queries. Within the subcategory of General Information there is another subcategory of Cancer Type which accounts for 427 queries or 32.28% of all General Information queries. Included in the fourth column underneath this relationship map are examples of actual user queries for the terms on the left. Neither spelling, nor punctuation nor capitalization has been corrected. These and all queries are taken directly from the logs, with the goal of illuminating the types of queries that the users are asking. Sometimes users type full, even excessively-long queries, and other times, they choose to use keywords.

It might not be possible to strictly compare categories for one Cancer Type to another because each analysis is driven by the user queries themselves. If 50% of all users asking about Breast Cancer had asked about Treatment, but no one querying Lung Cancer asked about treatment, there would be no Treatment subcategory under Lung Cancer.

Excerpted example illustrative of table contents (see explanation above, in Tables)

**Table A1:** General Information

Brain & Neurological 1852 - General Information 1323 72% Cancer Type 427 32.28%
how can I get information on glioblastoma
Astrocytoma Brain Tumor Research Online
find information on medulloblastoma
BENIGN BRAIN TUMORS

### Bone Cancer

**Table A2:** Bone Cancer

	Bone Cancer Total Count 1429
General Information	Where is information on bone cancer?
	What are the symptoms of bone cancer in teenagers?
Diagnosis &Testing	What is a bone marrow biopsy?
	What is the life expectancy of someone diagnosed with bone cancer?
	where find bone cancer treatments?
	how to deal with bone cancer pain?
	will agent orange cause bone cancer?
	bone cancer and the american cancer society
	which u.s. president had cancer in his left jaw?
	what are good web sites to look up bone cancer?
	prevention of bone cancer?
