# Supplementary material for: Searching for Cancer Information on the Internet: Analyzing Natural Language Search Queries
Source: J Med Internet Res. 2003 Dec 11;5(4):e31. doi: 10.2196/jmir.5.4.e31 (PMC1550578; doi:10.2196/jmir.5.4.e31)
Supplement: Supplementary file 1 [file jmir_v5i4e31_app1.zip › JMIR-2003-12-CE Appendix 1 copyedited.doc]

Appendix 1: Data Categorization, Counts, and Charts

Overview

Appendix 1 contains the counts and exact wording for all of the categorized questions from the sample of 7,500 user questions. Each category (such as *Breast or Head & Neck*) will have the highest level of breakdown on the first page, and subsequent breakdowns (if possible) on that page and following pages.

Rounding in Pie Charts

When looking at certain pie charts in Appendix 1 there will be categories that are shown to be 0%. This is due to rounding of numbers in Microsoft Excel. The actual percentage can be seen in the tabular format.

Additional Information in Pie Charts

The charts embedded within the Appendix can be double clicked to reveal additional information.

Tables

Most categories are broken out into tables with 4 columns. An excerpted example is shown below before the actual data tables are displayed. It shows the breakdown of Brain and Neurological Cancer > General Information > Cancer Type. The columns contain the following information:

- The first column starts with the category name in a yellow cell. In the example shown this is Cancer Type. Below Cancer Type are the types of cancers found within that category. For other categories, these would be the representative terms for that category, ie, for a category such as Treatment there might be listings for Alternative, Chemotherapy, Surgery, and Radiation.
- The second column of the illustrative table contains the raw count of user queries for that field. As shown in the example, Astrocytoma was queried 144 times, which is 33.72% of all queries that are found in the subcategory of Cancer Type.
- The third column shows the percentages for that subcategory. These are category specific, meaning that they are percentages of only those terms within that category or subcategory. Therefore Benign cancers represent 2.81% of all Cancer Type queries and are not 2.81% of all Brain & Neurological Cancer queries. While it was not the intention to include benign queries in this analysis, a small number were captured and analyzed, and therefore appear in the tables.
- The fourth column header notes where the subcategory is in relation to the main category. In this case Cancer Type was created within the General Information category of the cancer site Brain & Neurological Cancers. The counts are also included, to illustrate that out of the total number of Brain & Neurological Cancers (N = 1852 queries), General Information queries accounted for 1323 queries, which were 72% of all Brain and Neurological Cancer queries. Within the subcategory of General Information there is another subcategory of Cancer Type which accounts for 427 queries or 32.28% of all General Information queries. Included in the fourth column underneath this relationship map are examples of actual user queries for the terms on the left. Neither spelling, nor punctuation nor capitalization has been corrected. These and all queries are taken directly from the logs, with the goal of illuminating the types of queries that the users are asking. Sometimes users type full, even excessively-long queries, and other times, they choose to use keywords.

It might not be possible to strictly compare categories for one Cancer Type to another because each analysis is driven by the user queries themselves. If 50% of all users asking about Breast Cancer had asked about Treatment, but no one querying Lung Cancer asked about treatment, there would be no Treatment subcategory under Lung Cancer.

# Excerpted example illustrative of table contents (see explanation above, in Tables)

| **Cancer Type** | Count | Percent | **Brain & Neurological 1852 – General Information 1323 72%**  **– Cancer Type 427 32.28%** |
| --- | --- | --- | --- |
| Glioblastoma | 253 | 59.25% | how can I get information on glioblastoma |
| Astrocytoma | 144 | 33.72% | Astrocytoma Brain Tumor Research Online |
| Medulloblastoma | 18 | 4.22% | find information on medulloblastoma |
| Benign | 12 | 2.81% | BENIGN BRAIN TUMORS |

# Bone Cancer

| **BONE** | **Count** | **Percent** | **Bone Cancer Total Count 1429** |
| --- | --- | --- | --- |
| General Information | 1107 | 77.47% | Where is information on bone cancer? |
| Symptoms | 135 | 9.45% | What are the symptoms of bone cancer in teenagers? |
| Diagnosis & Testing | 64 | 4.48% | What is a bone marrow biopsy? |
| Statistics | 32 | 2.24% | What is the life expectancy of someone diagnosed with bone cancer? |
| Treatment | 26 | 1.82% | where find bone cancer treatments? |
| Coping - Pain | 23 | 1.61% | how to deal with bone cancer pain? |
| Cause/Risk/Link | 14 | 0.98% | will agent orange cause bone cancer? |
| Organization | 9 | 0.63% | bone cancer and the american cancer society |
| Celebrity | 8 | 0.56% | which u.s. president had cancer in his left jaw? |
| Websites | 7 | 0.49% | what are good web sites to look up bone cancer? |
| Prevention | 4 | 0.28% | prevention of bone cancer? |

| **General Information** | **Count** | **Percent** | **Bone Cancer 1429 – General Information 1107 77.47%** |
| --- | --- | --- | --- |
| Unspecified | 636 | 57.45% | Where can I find information about bone cancer? |
| Anatomy | 195 | 17.62% | Where can I find information on spinal cancer? |
| Ewings Sarcoma | 116 | 10.48% | Where can i find out about ewings sarcoma? |
| Definition | 74 | 6.68% | What is bone cancer? |
| Metastasize | 68 | 6.14% | What happens when cancer on the bone metastasizes? |
| Pictures | 18 | 1.63% | Where can i find pictures of bone cancer |

| **Diagnosis & Testing** | **Count** | **Percent** | **Bone Cancer 1429 – Diagnosis & Testing 64 4.48%** |
| --- | --- | --- | --- |
| Biopsy | 27 | 42.19% | how do i perform a bone marrow biopsy |
| CAT/CT Scan | 20 | 31.25% | diagnos Bone Ct Scan online |
| MRI | 13 | 20.31% | Where can I find MRI images of bone cancer |
| Unspecified | 4 | 6.25% | diagnosis of bone cancer |

| **Anatomy (Overall for Bone Queries)** | **Count** | **Percent** | **Bone Cancer 1429 – Anatomy 195 17.62%** |
| --- | --- | --- | --- |
| Back/Spine | 163 | 83.59% | Where can I find information on spinal cancer? |
| Jaw | 14 | 7.18% | Cancer Of Jawbone |
| Arm | 8 | 4.10% | Forearm Bone Cancer |
| Skull | 4 | 2.05% | find informati0n on skull tumors |
| Ribs | 4 | 2.05% | need informtion on bone cancer in rib cage |
| Leg | 2 | 1.03% | Bone cancer in the leg |

Brain & Neurological Cancer

| **Brain & Neurological** | **Count** | **Percent** | **Brain & Neurological Cancer Total Count 1852** |
| --- | --- | --- | --- |
| General Information | 1323 | 71.44% | where can i find information about brain cancer? |
| Symptoms | 259 | 13.98% | what are the symptoms of brain cancer? |
| Treatment | 112 | 6.05% | Brain Cancer Treatments Including Marijuana |
| Definition | 63 | 3.40% | what is brain cancer? |
| Cause/Risk/Link | 36 | 1.94% | can english muffins cause brain cancer? |
| Diagnosis & Testing | 22 | 1.19% | where can i get the CT scans of brain? |
| Statistics | 18 | 0.97% | what is brain cancer survival rate? |
| Coping-Support Group | 12 | 0.65% | Glioblastoma Survivors Support Groups |
| Organization | 7 | 0.38% | American Brain Tumor Association |

| **General Information** | **Count** | **Percent** | **Brain & Neurological 1852**  **– General Information 1323 71.44%** |
| --- | --- | --- | --- |
| Unspecified | 799 | 60.39% | where can i find information about brain cancer/ |
| Cancer Type | 427 | 32.28% | Where can I find info on glioblastoma? |
| Pictures | 57 | 4.31% | Where can i find pictures of brain cancer? |
| Types | 16 | 1.21% | How many kinds of brain cancer? |
| Metastasize | 12 | 0.91% | metastisized cancer brain |
| Websites | 8 | 0.60% | websites on cancerous tumors of the brain |
| History | 4 | 0.30% | where can i find history on brain cancer? |

| **Cancer Type** | **Count** | **Percent** | **Brain & Neurological 1852 – General Information 1323 72% – Cancer Type 427 32.28%** |
| --- | --- | --- | --- |
| Glioblastoma | 253 | 59.25% | how can I get information on glioblastoma |
| Astrocytoma | 144 | 33.72% | Astrocytoma Brain Tumor Research Online |
| Medulloblastoma | 18 | 4.22% | find information on medulloblastoma |
| Benign | 12 | 2.81% | BENIGN BRAIN TUMORS |

**Breast Cancer**

| **Breast Cancer** | **Count** | **Percent** | **Breast Cancer Total Count 6953** |
| --- | --- | --- | --- |
| General Information | 3423 | 49.23% | Where can I find information on breast cancer? |
| Symptoms | 889 | 12.79% | What are some symptoms of breast cancer? |
| Treatment | 570 | 8.20% | Where can I find information on breast cancer treatments? |
| Media/Organization | 428 | 6.16% | Where can I find information on the Avon Breast Cancer walk? |
| Cause/Risk/Link | 393 | 5.65% | what is the cause of breast cancer? |
| Diagnosis & Testing | 376 | 5.41% | how can i identify breast cancer in myself |
| Statistics | 274 | 3.94% | What age range is most affected by Breast Cancer? |
| Pictures | 225 | 3.24% | where can i find brest cancer pictures? |
| Type | 217 | 3.12% | where can I find out about a specific form of breast cancer? |
| Definition | 158 | 2.27% | what is breast cancer? |

| **General Information** | **Count** | **Percent** | **Breast Cancer 6953 – General Information 3423 49.23%** |
| --- | --- | --- | --- |
| Unspecified | 2868 | 83.79% | Where can I find information on breast cancer? |
| Male | 189 | 5.52% | where can I learn about breast cancer in men? |
| Stages | 164 | 4.79% | What is the difference between stage 2 and stage 3 breast cancer? |
| Coping - Support Groups | 87 | 2.54% | Where can I find support groups in my area/online? |
| Research/Journals | 84 | 2.45% | wher can i read recent research articles on breast cancer. |
| History | 13 | 0.38% | Where can I find information on history of breast cancer? |
| Celebrity | 8 | 0.23% | which celebrities have breast cancer |
| Clinical Trials | 6 | 0.18% | what are the top breast cancer hospitals |
| Pregnancy | 4 | 0.12% | what are the complications with breast cancer and pregnancy? |

| **General Information - Research** | **Count** | **Percent** | **Breast Cancer 6953 – General Information 3423 49% – Research 84 2.45%** |
| --- | --- | --- | --- |
| Unspecified | 22 | 26.19% | breast cancer research |
| Funding | 17 | 20.24% | breast cancer stastical research funding |
| Journals | 16 | 19.05% | Do you have a journal article on breast cancer? |
| Estrogen | 10 | 11.90% | ROLE OF ESTROGENS IN THE ACTIVITY OF BREAST CANCER |
| Local | 8 | 9.52% | 23rd Annual San Antonio Breast Cancer Symposium |
| Studies | 7 | 8.33% | breast cancer studies |
| Latest | 4 | 4.76% | How can I find out the latest on breast cancer research? |

| **Media/Organization** | **Count** | **Percent** | **Breast Cancer 6953 - Media/Organization 428 6.16%** |
| --- | --- | --- | --- |
| Awareness week/month | 94 | 21.96% | when is breast cancer awareness month? |
| Websites | 76 | 17.76% | What are the best sites about breast cancer? |
| Pins | 73 | 17.06% | where can I find breast cancer awareness pins |
| Events | 60 | 14.02% | Where can I find information on the Avon Breast Cancer walk? |
| Local | 42 | 9.81% | Bay Area Breast CAncer Network |
| Am. Breast Cancer Soc. | 23 | 5.37% | american breast cancer society |
| Ribbon | 22 | 5.14% | wher can I find a JPEG FILE OF THE BREAST CANCER RIBBON? |
| Stamp | 20 | 4.67% | Who sells breast cancer stamps |
| Charities | 12 | 2.80% | find breast cancer charities |
| Nat'l Breast Cancer Coalition | 6 | 1.40% | what is the National Breast Cancer Coalition |

| **Type** | **Count** | **Percent** | **Breast Cancer 6953 – Cancer Type 217 3.12%** |
| --- | --- | --- | --- |
| Recurrent | 62 | 28.57% | where can i find out about recurrent breast cancer |
| Metastastic | 46 | 21.20% | what is the prognosis for breast cancer metastatic to bone? |
| Inflammatory | 43 | 19.82% | find info on inflammatory breast cancer |
| Infiltrating Ductal | 39 | 17.97% | What is infiltrating ductal carcinoma of the breast |
| Lobular | 16 | 7.37% | Lobular In Situ Breast Cancer |
| Pagets | 11 | 5.07% | Invasive Paget's breast Cancer" |

| **Statistics** | **Count** | **Percent** | **Breast Cancer 6953 – Statistics 274 3.94%** |
| --- | --- | --- | --- |
| Survival Rate | 87 | 31.75% | what is the survival rate for breast cancer |
| Unspecified | 74 | 27.01% | where can I find statistics about breast cancer? |
| Age | 70 | 25.55% | What age range is most affected by Breast Cancer? |
| African American | 16 | 5.84% | Where can i find infroamtion on breast cancer in women in Massachusetts? |
| Annual Report | 15 | 5.47% | Where can I find the 2001 Statistics on Breast Cancer. |
| UK | 12 | 4.38% | breast cancer mortalities in the uk |

| **Cause/Risk/Link** | **Count** | **Percent** | **Breast Cancer 6953 – Cause/Risk/Link 393 5.65%** |
| --- | --- | --- | --- |
| Cause/Link | 290 | 73.79% | The Causes Of Breast Cancer |
| Prevention | 61 | 15.52% | How can a person prevent breast cancer? |
| Risk | 42 | 10.69% | what is the chance of getting breast cancer |

| **Cause/Risk/Link - Cause** | **Count** | **Percent** | **Breast Cancer 6953 – Cause/Risk/Link 393 6% - Cause 290 73.79%** |
| --- | --- | --- | --- |
| Deodorant | 114 | 39.31% | Is there any link between use of anti-perspirants and breast cancer? |
| General | 102 | 35.17% | where can i find out the causes of breast cancer |
| Genes | 26 | 8.97% | is breast cancer hereditary? |
| Foods | 9 | 3.10% | soy and breast cancer links? |
| Hormones | 8 | 2.76% | How are hormones related to breast cancer? |
| Metals | 7 | 2.41% | Any link between aluminum hydrochloride & breast cancer? |
| Aspartame | 7 | 2.41% | what are the links between aspartame and breast cancer |
| Clothing | 5 | 1.72% | is there a link between bras and breast cancer |
| Abortion | 4 | 1.38% | is there a connection between abortion and breast cancer? |
| Caffeine | 4 | 1.38% | does caffine cause breast cancer? |
| Tobacco | 4 | 1.38% | is there a relationship between breast cancer and smoking |

| **Symptoms** | **Count** | **Percent** | **Breast Cancer 6953 – Symptoms 889 12.79%** |
| --- | --- | --- | --- |
| Unspecified | 781 | 87.85% | What are some symptoms of breast cancer? |
| Hot Flash | 28 | 3.15% | Breast Cancer Hot Flashes |
| Itching | 20 | 2.25% | Itching Breast Cancer |
| Cysts | 17 | 1.91% | Cancerous Breast Cysts |
| Lumps | 16 | 1.80% | what does a cancerous breast lump feel like |
| Metastasis | 10 | 1.12% | Symptoms Of Metastasis Of Breast Cancer |
| Pain | 9 | 1.01% | Information on chest pains and breast cancer |
| Depression | 4 | 0.45% | Find information on breast cancer depression |
| Male | 4 | 0.45% | Male Breast Cancer Symptoms |

| **Treatment** | **Count** | **Percent** | **Breast Cancer 6953 – Treatment 570 8.20%** |
| --- | --- | --- | --- |
| Unspecified | 233 | 40.88% | where are best treatments for breast cancer |
| Chemotherapy | 99 | 17.37% | How is Breast Cancer treated with chemotherapy? |
| Alternative | 72 | 12.63% | alternative medicine for breast cancer |
| Radiation Therapy | 51 | 8.95% | What happens after radiation therapy for breast cancer? |
| Surgery | 40 | 7.02% | total mastectomy an option for breast cancer with secondry cancer in lymph node? |
| Medications | 28 | 4.91% | breast cancer medications |
| Cure | 15 | 2.63% | is there a cure for breast cancer |
| Experimental | 14 | 2.46% | where can i find info on experimental breast cancer treatment |
| Latest | 10 | 1.75% | lastest treatment of breast cancer |
| Male | 8 | 1.40% | what is the treatment for male breast cancer |
| Latest | 10 | 1.75% | lastest treatment of breast cancer |
| Male | 8 | 1.40% | what is the treatment for male breast cancer |

| **Diagnosis & Testing** | **Count** | **Percent** | **Breast Cancer 6953 – Diagnosis & Testing 376 5.41%** |
| --- | --- | --- | --- |
| Biopsy | 153 | 40.69% | what is a breast ultra sound guided core biopsy |
| Self-Diagnosis | 92 | 24.47% | how can i identify breast cancer in myself |
| Diagnosis-Unspecified | 90 | 23.94% | how do u detect breast cancer |
| Markers | 16 | 4.26% | Breast Cancer Markers |
| Genetics | 7 | 1.86% | breast cancer DNA testing |
| Ultrasound | 6 | 1.60% | Breast Cancer Ultrasound |
| Mammogram | 4 | 1.06% | will a mammogram detect breast cancer? |
| Sonogram | 4 | 1.06% | sonogram breast cancer |
| UK | 4 | 1.06% | detcting breast cancer. u.k. |

**Cause & Risk**

| **Cause & Risk** | **Count** | **Percent** |
| --- | --- | --- |
| Causes and Links | 1115 | 89.27% |
| Prevention | 134 | 10.73% |
| Cause & Risk Total Count | 1249 |  |

| **Cause/Link** | **Count** | **Percent** | **Cause & Risk 1249 – Cause/Link 1115 89.27%** |
| --- | --- | --- | --- |
| Drugs | 287 | 25.74% | how does smoking cause cancer |
| Unspecified | 247 | 22.15% | what is cause a cancer? |
| Radiation | 247 | 22.15% | picture of cell phone radiation and cancer |
| Personal | 116 | 10.40% | Can anti-persperant deodorant cause cancer? |
| Chemicals/Plastics | 74 | 6.64% | Does microwaving plastic cause cancer? |
| Environmental | 70 | 6.28% | do power lines cause cancer? |
| Food Supplements | 64 | 5.74% | Can Creatine cause Cancer |
| Genetic Mutation/Virus | 10 | 0.90% | can a virus start carcinogenises? |

| **Prevention** | **Count** | **Percent** | **Cause & Risk 1249 – Prevention 134 10.73%** |
| --- | --- | --- | --- |
| Unspecified | 84 | 62.69% | how to prevent cancer |
| Nutrition/Diet | 29 | 21.64% | what foods prevent cancer? |
| Alternative | 12 | 8.96% | How do antioxidants prevent cancer that may be caused by free radicals? |
| Exercise | 9 | 6.72% | Can excercise prevent of cancer? |

| **Drugs** | **Count** | **Percent** | **Cause & Risk 1249 – Cause/Link 1115 89% - Drugs 287 25.74%** |
| --- | --- | --- | --- |
| Tobacco | 138 | 48.08% | How is tobacco use associated with cancer? |
| Marijuana | 62 | 21.60% | does smoking marijuana cause cancer |
| Alcohol | 49 | 17.07% | Can alcohol cause cancer? |
| Medication | 18 | 6.27% | can aspirin cause cancer? |
| Contraceptives | 13 | 4.53% | Do oral contraceptives cause cancer |
| Unspecified | 7 | 2.44% | Where can I find information about cancers caused by drugs? |

| **Environmental** | **Count** | **Percent** | **Cause & Risk 1249 – Cause/Link 1115 89% - Environmental 70 6.28%** |
| --- | --- | --- | --- |
| Unspecified | 30 | 42.86% | What are the environmental facters that causecancer? |
| Tanning Beds | 10 | 14.29% | Can tanning beds cause cancer? |
| Clothing | 9 | 12.86% | can underwire bras cause cancer? |
| Power Lines | 8 | 11.43% | Does High Power Lines cause cancer? |
| Plants | 7 | 10.00% | are ferns carcinogenic |
| Smog | 6 | 8.57% | does air pollution cause cancer? |

| **Food Supplements** | **Count** | **Percent** | **Cause & Risk 1249 – Cause/Link 1115 89% - Food Supp. 64 5.74%** |
| --- | --- | --- | --- |
| Creatine | 25 | 39.06% | does taking creatine cause cancer? |
| Vitamins | 20 | 31.25% | Can Vitamin C cause cancer? |
| Aspartame | 11 | 17.19% | Does aspartame cause cancer? |
| Food Dyes | 8 | 12.50% | does yellow 5 cause cancer? |

| **Radiation** | **Count** | **Percent** | **Cause & Risk 1249 – Cause/Link 1115 89% - Radiation 247 22.15%** |
| --- | --- | --- | --- |
| Cell Phone | 189 | 77.14% | do cellular phones cause cancer |
| General | 58 | 22.86% | Linds between cancer and electromagnetic radiation |

| **Personal** | **Count** | **Percent** | **Cause & Risk 1249 – Cause/Link 1115 89% - Personal 116 10.40%** |
| --- | --- | --- | --- |
| Hair Dyes | 48 | 41.38% | hair dye and cancer |
| Deodorant | 36 | 31.03% | does anti-perspirant cause cancer |
| Foods/Nutrition | 20 | 17.24% | why does vegetable fiber increase the risk of cancer in the nurses health study |
| Menopause | 5 | 4.31% | cancers caused by menopause |
| Muscle Sprain | 4 | 3.45% | can sprained muscles cause cancer |
| Stress | 3 | 2.59% | does stress cause cancer |

# Coping

| **Coping** | **Count** | **Percent** | **Coping Total Count 254** |
| --- | --- | --- | --- |
| Support Groups | 127 | 50.00% | cancer support group |
| Pain | 98 | 38.58% | where can i find information on pain managemnet for cancer |
| Depression | 29 | 11.42% | depression from cancer |

Diagnosis & Testing

| **Diagnosis & Testing** | **Count** | **Percent** | **Diagnosis & Testing Total Count 3315** |
| --- | --- | --- | --- |
| Testing | 2842 | 85.73% | what does cat scans show |
| Symptoms | 473 | 14.27% | what are some symptoms of cancer |

| **Testing** | **Count** | **Percent** | **Diagnosis & Testing 3315 – Testing 2842 85.73%** |
| --- | --- | --- | --- |
| CAT/CT Scan | 1509 | 53.10% | how cat scans work |
| MRI | 587 | 20.65% | Explain how Magnetic Resonance Imaging detects cancer |
| Biopsy | 502 | 17.66% | what is a stereotactic biopsy? |
| Blood | 128 | 4.50% | blood tests for cancer |
| Multiple | 54 | 1.90% | What is the difference between a CAT scan and an MRI? |
| General Information | 51 | 1.79% | What tests are used for cancer |
| PET Scan | 11 | 0.39% | What is a PET test for cancer? |

| **CAT/CT Scan** | **Count** | **Percent** | **Diagnosis & Testing 3315 – Testing 2842 85.73% – CAT/CT Scan 1509 53.10%** |
| --- | --- | --- | --- |
| Unspecified | 643 | 42.61% | where can i find information on cat scans |
| What is….? | 430 | 28.50% | what is a cat scan |
| Anatomy | 124 | 8.22% | Pelvic cat scan |
| Safety | 59 | 3.91% | is a cat scan safe? |
| How does it work? | 48 | 3.18% | how does a cat scan work |
| Images/Pictures | 46 | 3.05% | where can i see CT scan pictures? |
| Full Body | 41 | 2.72% | Ct Scan Full Body |
| Spiral Scan | 21 | 1.39% | what is a spiral cat scan |
| Costs | 18 | 1.19% | How much does a CAT scan cost? |
| (Iodine) Contrast | 16 | 1.06% | CT scan with contrast |
| Who invented…? | 16 | 1.06% | who invented the Ct Scan? |
| Acronym | 12 | 0.80% | What does CT scan stand for? |
| Location | 11 | 0.73% | where can i get a ct scan in los angeles |
| Specialists | 10 | 0.66% | information on a cat scan specialist |
| Mobile | 8 | 0.53% | mobile ct scan |
| Ultra Fast | 6 | 0.40% | where can I have an ultra fast CT scan done |

| **Biopsy** | **Count** | **Percent** | **Diagnosis & Testing 3315 – Testing 2842 85.73% - Biopsy 502 17.66%** |
| --- | --- | --- | --- |
| Unspecified | 246 | 49.00% | where can i find infomation on biopsy |
| Cone | 67 | 13.35% | what is a cone biopsy |
| Anatomy | 60 | 11.95% | INTERCRANIAL BIOPSY |
| Stereotactic | 39 | 7.77% | what is a stereotactic biopsy? |
| Needle | 32 | 6.37% | fine needle aspiration biopsy stories |
| Safety | 24 | 4.78% | Is it safe to have a biopsy performed during pregnancy? |
| Punch | 15 | 2.99% | what is a punch biopsy? |
| Images/Pictures | 8 | 1.59% | biopsy pictures |
| Ultrasound | 8 | 1.59% | Ultra Sound Biopsy |
| Frozen | 3 | 0.60% | frozen section biopsy |

**Digestive – Gastrointestinal – Bowel**

| **Digestive/Gastrointestinal/Bowel - Overall** | **Count** | **Percent** |
| --- | --- | --- |
| General Information | 5568 | 62.15% |
| Symptoms | 1506 | 16.81% |
| Diagnosis & Testing | 1125 | 12.56% |
| Treatment | 294 | 3.28% |
| Statistics | 184 | 2.05% |
| Definition | 163 | 1.82% |
| Cause/Risk/Link | 119 | 1.33% |

| **Cause/Risk/Link** | **Count** | **Percent** | **Digestive – Gastrointestinal – Bowel 8959 – Cause/Risk/Link 119 1.33%** |
| --- | --- | --- | --- |
| Cause | 82 | 68.91% | what chemicals can cause pancreatic cancer? |
| Risk | 15 | 12.61% | am i at-risk for colon cancer |
| Prevention | 14 | 11.76% | how do i prevent colon cancer? |
| Link | 8 | 6.72% | relationship between alcohol and pancreatic cancer |

| **Diagnosis & Testing** | **Count** | **Percent** | **Digestive – Gastrointestinal – Bowel 8959**  **– Diagnosis & Testing 1125 12.56%** |
| --- | --- | --- | --- |
| Colonoscopy | 940 | 83.56% | Where can I find information regarding colonoscopys |
| Biopsy | 114 | 10.13% | how is a liver biopsy done |
| Testing-General | 29 | 2.58% | What are the recommended tests to screen for colorectal cancer? |
| CAT/CT Scan | 20 | 1.78% | ct scan with dye for colon diseases |
| Sigmoidoscopy | 15 | 1.33% | Colon Cancer Training Sigmoidoscopy |
| Fluoroscopy | 7 | 0.62% | Difference between colonoscopy and fluoroscopy? |

| **General Information** | **Count** | **Percent** |
| --- | --- | --- |
| Colorectal | 2611 | 46.89% |
| Liver | 985 | 17.69% |
| Gastrointestinal | 730 | 13.11% |
| Pancreas | 683 | 12.27% |
| Esophageal | 220 | 3.95% |
| Bowel | 191 | 3.43% |
| Bile | 66 | 1.19% |
| Gall Bladder | 41 | 0.74% |
| Anal | 33 | 0.59% |
| Abdominal | 8 | 0.14% |

| **Pancreas** | **Count** | **Percent** | **Digestive – Gastrointestinal – Bowel 8959 – Pancreas 965 10.77%** |
| --- | --- | --- | --- |
| General Information | 683 | 70.78% | When do people who have pancreatic cancer know that they have the disease? |
| Symptoms | 121 | 12.54% | What are the symptons of Pancreatic Cancer? |
| Treatment | 57 | 5.91% | Where can i learn about new treatments for pancreatic cancer |
| Cause/Risk/Link | 39 | 4.04% | what chemicals can cause pancreatic cancer? |
| Statistics | 32 | 3.32% | Mortality Of Pancreatic Cancer |
| Defintion | 25 | 2.59% | what is cancer of the pancreas |
| Diagnosis & Testing | 8 | 0.83% | Are there any test for cancer of the pancreas? |

| **Pancreas - Treatment** | **Count** | **Percent** | **Digestive – Gastrointestinal – Bowel 8959 – Pancreas 965 10.77% - Treatment 57 5.91%** |
| --- | --- | --- | --- |
| Alternative | 26 | 45.61% | Where can I find information on holistic medicine to treat pancreatic cancer |
| Unspecified | 18 | 31.58% | What is the best treatment for pancreatic cancer? |
| Latest | 8 | 14.04% | Are there any promising breakthroughs for pancreatic cancer? |
| Cure | 4 | 7.02% | Are there any studies which may prove to cure pancreatic cancer? |
| Hormone | 1 | 1.75% | how are hormones used to treat cancer of the pancreas |

| **Bowel Cancer** | **Count** | **Percent** | **D/G/B 8959 – Bowel Cancer 273 3.05%** |
| --- | --- | --- | --- |
| General Information | 191 | 69.96% | information on bowel cancer |
| Symptoms | 60 | 21.98% | signs of bowel cancer |
| Definition | 14 | 5.13% | what is bowel cancer? |
| Treatment | 4 | 1.47% | trearments for bowel cancer |
| Statistics | 4 | 1.47% | statistics on bowel cancer in australia |

| **Colorectal** | **Count** | **Percent** | **Digestive – Gastrointestinal – Bowel 8959 – Colorectal 4801 53.58%** |
| --- | --- | --- | --- |
| General Information | 2611 | 54.38% | where can i find information on colon cancer? |
| Diagnosis & Testing | 997 | 20.77% | How is a colonoscopy performed |
| Symptoms | 979 | 20.39% | What are symptoms of colon cancer? |
| Treatment | 78 | 1.62% | new medication for colon cancer |
| Definition | 57 | 1.19% | what is cancer of the rectum? |
| Statistics | 42 | 0.87% | What is the survival rate for colon cancer? |
| Cause/Risk/Link | 37 | 0.77% | what causes colon cancer |

| **Esophageal** | **Count** | **Percent** | **Digestive – Gastrointestinal – Bowel 8959 – Esophageal 260 2.90%** |
| --- | --- | --- | --- |
| General Information | 220 | 84.62% | Where can I learn about the cancer esophageal cancer? |
| Treatment | 12 | 4.62% | esophageal cancer treatments |
| Sympoms | 10 | 3.85% | esophageal cancer symptoms |
| Statistics | 10 | 3.85% | ESOPHAGEAL CANCER SURVIVAL STATISTICS |
| Cause/Risk/Link | 7 | 2.69% | what are the risks of phemonia with esophageal cancer? |
| Definition | 1 | 0.38% | what is esophageel cancer? |

| **Gastrointestinal** | **Count** | **Percent** | **Digestive – Gastrointestinal – Bowel 8959 – Gastrointestinal 1094 12.21%** |
| --- | --- | --- | --- |
| General Information | 730 | 66.73% | Where can I find information on stomach cancer? |
| Symptoms | 220 | 20.11% | what are the symtoms for stomach cancer? |
| Statistics | 58 | 5.30% | what country has a high rate of stomach cancer |
| Definition | 48 | 4.39% | what is intestinal cancer |
| Treatment | 20 | 1.83% | treatment of stage three stomach cancer |
| Cause/Risk/Link | 18 | 1.65% | what causes stomach cancer |

| **Gastrointestinal - General** | **Count** | **Percent** | **D/G/B 8959 – Gastrointestinal 1094 12.21% - General Info 788 72.03%** |
| --- | --- | --- | --- |
| Unspecified | 696 | 88.32% | Where can I find information on stomach cancer? |
| Statstics | 58 | 7.36% | does japan have a high rate of stomach cancer |
| Stages | 24 | 3.05% | stage 4 stomach cancer |
| Websites | 10 | 1.27% | where can I find a website on stomach cancer |

| **Colorectal - Diagnosis** | **Count** | **Percent** | **Digestive – Gastrointestinal – Bowel 8959 – Colorectal 4801 53.58% - Diagnosis 997 20.77%** |
| --- | --- | --- | --- |
| Colonoscopy | 940 | 94.28% | How do prepare for a colonoscopy? |
| Testing-General | 21 | 2.11% | diagnosis of colorectal cancer |
| Sigmoidoscopy | 15 | 1.50% | How does sigmoidoscopy differ from colonoscopy |
| Cat/CT scan | 10 | 1.00% | ct scan with dye for colon diseases |
| Fluoroscopy | 7 | 0.70% | Difference between colonoscopy and fluoroscopy? |
| Biopsy | 4 | 0.40% | colonoscopy polypectomy biopsp |

| **Liver** | **Count** | **Percent** | **Digestive – Gastrointestinal – Bowel 8959 – Liver 1413 15.77%** |
| --- | --- | --- | --- |
| General Information | 985 | 70.11% | Where can I find information on liver cancer? |
| Treatment | 128 | 8.54% | What are treatment for liver cancer |
| Diagnosis & Testing | 116 | 8.26% | information about liver biopsy |
| Symptoms | 116 | 8.26% | what are symptoms of liver cancer |
| Statistics | 38 | 2.70% | how many people get liver cancer every year |
| Cause/Risk/Link | 18 | 1.28% | what are the risk factors of liver cancer? |
| Definition | 12 | 0.85% | what is liver cancer? |

| **Liver - General Information** | **Count** | **Percent** | **Digestive – Gastrointestinal – Bowel 8959 – Liver 1413 15.77% - General Info 985 69.70%** |
| --- | --- | --- | --- |
| Unspecified | 863 | 87.61% | Where can I find information on liver cancer? |
| Metastasize | 76 | 7.72% | Metastatic Liver Cancer |
| Secondary | 22 | 2.23% | where to find secondary liver cancer |
| Stages | 12 | 1.22% | stage 4 liver cancer |
| Clinical Trials | 8 | 0.81% | clinical trials for liver cancer |
| Celebrity | 4 | 0.41% | Does Courtney Cox have liver cancer? |

| **Liver - Treatment** | **Count** | **Percent** | **Digestive – Gastrointestinal – Bowel 8959 – Liver 1413 15.77% - Treatment 128 9.05%** |
| --- | --- | --- | --- |
| Unspecified | 74 | 57.81% | what treatments are possible for liver cacner |
| Alternative | 10 | 7.81% | herbal treatment for liver cancer |
| Radiation Therapy | 10 | 7.81% | radiofrequency ablation for liver cancer |
| Clinical Trials | 8 | 6.25% | clinical trials for liver cancer |
| Cure | 8 | 6.25% | cures for liver cancer metasis |
| Steroids | 8 | 6.25% | Steroid Treatments For Liver Cancer |
| Surgery | 4 | 3.13% | laser surgery for secondary liver cancer |
| Transplant | 4 | 3.13% | Liver Transplants - Cancer |
| Latest | 2 | 1.56% | where can I find the latest treatments for liver cancer |

**General Research**

| **General Research** | **Count** | **Percent** | **General Research Total Count 7808** |
| --- | --- | --- | --- |
| Research | 2819 | 36.10% | Where can I find out the latest on cancer research? |
| Organization | 1656 | 21.21% | where can i find information on the american cancer society |
| Clinical Trials | 1272 | 16.29% | Where can I find info on FDA approved clinical trials. |
| Concerns | 1201 | 15.38% | how can i donate my hair to cancer |
| Pictures | 559 | 7.16% | Where can I find pictures of cancer? |
| Websites | 194 | 2.48% | Where can I find web pages about cancer |
| History | 66 | 0.85% | History of the search for the cure for cancer |
| Statistics | 23 | 0.29% | How many deaths does cancer cause in the United States? |
| Legal/Lawyers | 14 | 0.18% | NY STATE LAW CONCERNING TREATMENT OF BREAST CANCER |
| Lesson Plans | 4 | 0.05% | cancer lesson plans |

| **Concerns** | **Count** | **Percent** | **General Research 7808 – Concerns 1201 15.38%** |
| --- | --- | --- | --- |
| Pets | 861 | 71.69% | what are the most common tumors in dogs |
| Donate Hair | 274 | 22.81% | find out how to give hair to a cancer foundation so they can make a wig out of it? |
| Celebrity | 58 | 4.83% | what kind of cancer did lance armstrong have? |
| AIDS | 8 | 0.67% | can people with cancer get aids |

| **Organization** | **Count** | **Percent** | **General Research 7808 – Organization 1656 21.21%** |
| --- | --- | --- | --- |
| ACS | 1065 | 64.31% | where can i find information from american cancer society |
| NCI | 223 | 13.47% | where can I find the national cancer institute? |
| State/Local | 132 | 7.97% | where is the best cancer center in texas |
| International | 106 | 6.40% | cancer institutues in Mexico |
| ACA | 66 | 3.99% | information of american cancer association |
| NCS | 31 | 1.87% | where is the National Cancer Society |
| CSA | 12 | 0.72% | Where can I find the cancer society of america homepage? |
| RPCI | 9 | 0.54% | roswell park cancer institute |
| CFOA | 4 | 0.24% | Cancer Fund of America phone number |
| ISOC | 4 | 0.24% | International Society of Chemotherapy |
| JWCI | 4 | 0.24% | John Wayne Cancer Institute |

| **Clinical Trial** | **Count** | **Percent** | **General Research 7808 - Clinical Trials 1272 16.29%** |
| --- | --- | --- | --- |
| What are… | 634 | 49.84% | What are clinical trials? |
| Latest | 260 | 20.44% | latest cancer clinical trial research |
| Type of… | 111 | 8.73% | types of cancer trials |
| Medical Condition | 64 | 5.03% | Where can I learn about the results of clinical trials to cure diabetes?? |
| Run by… | 59 | 4.64% | Where can I find information on NIH clinical trials? |
| Medications | 44 | 3.46% | please show me trial studies using the drug c255 for cancer |
| State/Local | 29 | 2.28% | what research and clinical trials are in nyc at this time |
| FDA Approved | 21 | 1.65% | Where can I find info on FDA approved clinical trials. |
| History | 20 | 1.57% | history of clinical trials |
| Year Results | 14 | 1.10% | How many clinical trials were conducted in 2000? |
| Web-Based | 12 | 0.94% | Web-based Clinical Trials |
| Publications | 4 | 0.31% | Who wrote the book Fundamentals of Clinical Trials? When was it published? |

**Genitourinary**

| **Genitourinary** | **Count** | **Percent** | **Genitourinary Total Count 6250** |
| --- | --- | --- | --- |
| General Information | 3762 | 60.19% | info in prostate cancer |
| Symptoms | 854 | 13.66% | What are symptoms of testicular cancer? |
| Treatment | 604 | 9.66% | where can i find information on prostrate cancer and seed implantationtherapy |
| Definitions | 365 | 5.84% | What is genitourinary cancer? |
| Diagnosis & Testing | 294 | 4.70% | How can I do a self-check to see if I have testicular cancer? |
| Pictures | 121 | 1.94% | where can i find pictures of prostate cancer? |
| Statistics | 74 | 1.18% | testicular cancer motality rates in great britain |
| Coping | 64 | 1.02% | prostate cancer support chat |
| Cause/Risk/Link | 62 | 0.99% | what are the causes of prostate cancer? |
| Concerns - Celebrity | 19 | 0.30% | Famous People with Prostate Cancer |
| Prevention | 18 | 0.29% | How do you prevent prostate cancer? |
| Organization | 13 | 0.21% | prostate cancer society |

| **Symptoms** | **Count** | **Percent** | **Genitourinary 6250 – Symptoms 854 13.66%** |
| --- | --- | --- | --- |
| Testicular | 378 | 44.26% | Where can I learn about symptoms and facts on testicular cancer? |
| Prostate | 364 | 42.62% | signs and symptoms of prostate cancer in young men |
| Bladder | 81 | 9.48% | what are symptoms of bladder cancer? |
| Penile | 18 | 2.11% | what are the symtons of cancer of the penis |
| Kidney | 13 | 1.52% | symptoms of kidney cancer |

| **General Information** | **Count** | **Percent** | **Genitourinary 6250 – General Information 3762 60.19%** |
| --- | --- | --- | --- |
| Prostate | 1716 | 45.61% | can you have prostate cancer with out a prostate? |
| Testicular | 1058 | 28.12% | WHere can I find info on testicular cancer? |
| Bladder | 506 | 13.45% | where can i find information about male bladder cancer ? |
| Kidney | 383 | 10.18% | where can i find information on kidney cancer |
| Penile | 99 | 2.63% | Where can I learn about penile cancer? |

| **Treatment** | **Count** | **Percent** | **Genitourinary 6250 – Treatment 604 9.66%** |
| --- | --- | --- | --- |
| Unspecified | 397 | 65.73% | Where can I find information relating to the treatment of Bladder Cancer? |
| Radiation Therapy | 72 | 11.92% | where can I find information about prostate cancer and seed implantation |
| Alternative | 50 | 8.28% | where can i find alternative treatments for bladder cancer |
| Surgery | 32 | 5.30% | prostate cancer surgery |
| Cures | 15 | 2.48% | Is there a cure for kidney cancer? |
| Medications | 15 | 2.48% | Prostate cancer treatment+chemotherapy |
| BCG | 6 | 0.99% | Bacillus Calmette-guerin Bladder Cancer Treatment |
| Freezing | 5 | 0.83% | prostate cancer freezing Orlando |
| Hormone | 4 | 0.66% | What are the side effects of hormone treatments for Prostate cancer? |
| Immunotherapy | 4 | 0.66% | Bladder Cancer Immunotherapy |
| TARGIS | 4 | 0.66% | where can I find information about the TARGIS system for prostate cancer? |

| **Bladder** | **Count** | **Percent** | **Genitourinary 6250 – Bladder 708 11.33%** |
| --- | --- | --- | --- |
| General Information | 506 | 71.47% | where can I find information on bladder cancer? |
| Symptoms | 81 | 11.44% | What are the signs of bladder cancer |
| Treatment | 78 | 11.02% | where can i find alternative treatments for bladder cancer |
| Definition | 24 | 3.39% | What is bladder cancer? |
| Cause/Risk/Link | 11 | 1.55% | WHAT CAUSES BLADDER CANCER |
| Statistics | 8 | 1.13% | statistics on bladder cancer |

| **Kidney** | **Count** | **Percent** | **Genitourinary 6250 – Kidney 496 7.94%** |
| --- | --- | --- | --- |
| General Information | 383 | 77.22% | Where can I learn about the kidney or urinary tract condition kidney (renal) cancer? |
| Diagnosis & Testing | 43 | 8.67% | wHAT IS A RENAL BIOPSY |
| Treatment | 34 | 6.85% | WHERE CAN I FIND TREATMENT OPTIONS FOR RENAL CARCINOOMA? |
| Symptoms | 13 | 2.62% | what are the symptoms of kidney cancer? |
| Definition | 12 | 2.42% | What is kidney cancer? |
| Statistics | 4 | 0.81% | kidney cancer survival rate |
| Organization | 4 | 0.81% | donating my car to the kidney foundation or cancer society |
| Cause/Risk/Link | 3 | 0.60% | What causes a tumor on your kidneys? |

| **Prostate** | **Count** | **Percent** | **Genitourinary 6250 – Prostate 3141 50.26%** |
| --- | --- | --- | --- |
| General Information | 1715 | 54.60% | Where can I find information about prostate cancer? |
| Treatment | 460 | 14.65% | Information about prostate cancer and treatments |
| Symptoms | 364 | 11.59% | Where can I find the symptoms of prostate cancer? |
| Definition | 246 | 7.83% | what is prostate cancer? |
| Diagnosis & Testing | 150 | 4.78% | will an mri detect prostate cancer |
| Statistics | 48 | 1.53% | Howmany men die a year do to prostate cancer? |
| Coping | 42 | 1.34% | Is there a sex life after prostrate cancer? |
| Cause/Risk/Link | 40 | 1.27% | what are the causes of prostate cancer? |
| Pictures | 30 | 0.96% | where can i find pictures of prostate cancer? |
| Prevention | 18 | 0.57% | How do you prevent prostate cancer? |
| Organization | 9 | 0.29% | prostate cancer society |
| Concerns/Celebrity | 19 | 0.60% | Famous People with Prostate Cancer |

| **Testicular** | **Count** | **Percent** | **Genitourinary 6250 – Testicular 1772 28.35%** |
| --- | --- | --- | --- |
| General Information | 1059 | 59.76% | What should I know about testicle cancer? |
| Symptoms | 378 | 21.33% | Where can I learn about symptoms and facts on testicular cancer? |
| Diagnosis & Testing | 97 | 5.47% | How can I do a self-check to see if I have testicular cancer? |
| Pictures | 87 | 4.91% | pictures of testicular cancer cells |
| Definition | 75 | 4.23% | what is testicle cancer? |
| Treatment | 32 | 1.81% | treatment for testicular cancer |
| Coping | 22 | 1.24% | is there pain in testicular cancer? |
| Statistics | 14 | 0.79% | How many men are diagnosed with testicular cancer annually? |
| Cause/Risk/Link | 8 | 0.45% | Is testiclular cancer inherited? |

| **Prostate Treatment** | **Count** | **Percent** | **Genitourinary 6250 – Prostate 3141 50.26% - Treatment 460 14.65%** |
| --- | --- | --- | --- |
| Unspecified | 287 | 62.39% | where can i find information on prostrate cancer therapy |
| Radiation Therapy | 72 | 15.65% | where can I find information about prostate cancer and seed implantation |
| Surgery | 32 | 6.96% | information on prostate cancer surgery |
| Alternative | 30 | 6.52% | what foods should i eat to fight prostate cancer |
| Cure | 11 | 2.39% | can prostate cancer be cured |
| Medications | 15 | 3.26% | Prostate cancer treatment+chemotherapy |
| Freezing | 5 | 1.09% | prostate cancer freezing Orlando |
| Hormone | 4 | 0.87% | What are the side effects of hormone treatments for Prostate cancer? |
| Targis | 4 | 0.87% | where can I find information about the TARGIS system for prostate cancer? |

**Gynecological**

| **Gynecological** | **Count** | **Percent** | **Gynecological Total Count 5344** |
| --- | --- | --- | --- |
| General Information | 3409 | 63.79% | information on ovarian cancer |
| Symptoms | 939 | 17.57% | what are the symptoms of ovarian cancer |
| Diagnosis & Testing | 452 | 8.46% | How can I find out information on vulva biopsy |
| Treatment | 247 | 4.62% | Where can i find out about the tradional treatments of cervical cancer? |
| Definition | 158 | 2.96% | what is cervical cancer |
| Cause/Risk | 83 | 1.55% | what causes cervical dysplasia |
| Statistics | 42 | 0.79% | What is the incidence of cervical cancer? |
| Prevention | 14 | 0.26% | Prevention of cervical cancer |

| **Cervical** | **Count** | **Percent** | **Gynecological 5344 – Cervical 1924 36.00%** |
| --- | --- | --- | --- |
| General Information | 1283 | 66.68% | where can I find information on cervical cancer? |
| Symptoms | 243 | 12.63% | symptoms of cervical cancer |
| Testing/Diagnosis | 116 | 6.03% | cervical biopsy |
| Treatment | 101 | 5.25% | cervical cancer treatments |
| Definition | 90 | 4.68% | what is cancer of the cervic? |
| Cause | 71 | 3.69% | causes of cervical cancer |
| Statistics | 16 | 0.83% | What is the incidence of cervical cancer? |
| Prevention | 4 | 0.21% | Prevention of cervical cancer |

| **Endometrial** | **Count** | **Percent** | **Gynecological 5344 – Endometrial 225 4.21%** |
| --- | --- | --- | --- |
| General Information | 100 | 44.44% | Where will I find information on endometrial cancer? |
| Testing/Diagnosis | 95 | 42.22% | where can I find informatin on endometrial biopsies |
| Symptoms | 26 | 11.56% | symptoms of endometrious cancer |
| Prevention | 4 | 1.78% | Does progresterone prevent endometrial cancer? |

| **Ovarian** | **Count** | **Percent** | **Gynecological 5344 – Ovarian 2031 38.00%** |
| --- | --- | --- | --- |
| General Information | 1284 | 63.22% | information on ovarian cancer |
| Symptoms | 525 | 25.85% | symptoms of ovarian cancer? |
| Treatment | 121 | 5.96% | What treatments are available for Ovarian Cancer? |
| Definition | 56 | 2.76% | what is ovarian cancer |
| Statistics | 15 | 0.74% | where can i find statistics about ovarian cancer? |
| Cause | 12 | 0.59% | What causes ovarian cancer |
| Testing/Diagnosis | 12 | 0.59% | blood test for ovarian cancer |
| Prevention | 6 | 0.30% | ovarian cancer screening prevention |

| **Uterine** | **Count** | **Percent** | **Gynecological 5344 – Uterine 606 11.34%** |
| --- | --- | --- | --- |
| General Information | 434 | 71.62% | what is uterine cancer? |
| Symptoms | 112 | 18.48% | symptoms of uterine cancer |
| Testing/Diagnosis | 35 | 5.78% | biopsy of the uterus |
| Treatment | 19 | 3.14% | where can i find information about hormone cancer therapy for uterine cancer? |
| Definition | 4 | 0.66% | what is uterine cancer? |
| Statistics | 2 | 0.33% | survival rate uterine cancer |

| **Vulvar** | **Count** | **Percent** | **Gynecological 5344 – Vulvar 166 3.11%** |
| --- | --- | --- | --- |
| General Information | 134 | 80.72% | cancer of vulva |
| Testing/Diagnosis | 13 | 7.83% | How can I find out information on vulva biopsy |
| Statistics | 9 | 5.42% | statistis on vulva cancer |
| Treatment | 6 | 3.61% | side effects of radiation for vulvar cancer? |
| Symptoms | 4 | 2.41% | symptoms of vulvar dysplasia |

| **Vaginal** | **Count** | **Percent** | **Gynecological 5344 – Vaginal 219 4.09%** |
| --- | --- | --- | --- |
| General Information | 163 | 74.43% | Information on Vaginal Cancer |
| Symptoms | 29 | 13.24% | vaginal cancer symptoms |
| Testing/Diagnosis | 19 | 8.68% | where can I find information on vaginal biopsy |
| Definition | 8 | 3.65% | what is vaginal cncer? |

| **General Information** | **Count** | **Percent** | **Gynecological 5344 – General Info 3409 63.79%** |
| --- | --- | --- | --- |
| Ovarian | 1284 | 37.67% | Tell me about ovarian cancer |
| Cervical | 1283 | 37.64% | Where can I get information on cervical cancer? |
| Uterine | 434 | 12.73% | Where can I find info re cancer of the uterus? |
| Vaginal | 163 | 4.78% | Is it true you can get cancer in the vagina? |
| Vulvar | 134 | 3.93% | cancer of the vulva |
| Endometrial | 100 | 2.93% | Where will I find information on endometrial cancer? |
| Gestational Trophoblastic | 11 | 0.32% | information about gesticular trophoblastic disease? |

| **Cause** | **Count** | **Percent** | **Gynecological 5344 – Cause 83 1.55%** |
| --- | --- | --- | --- |
| Unspecified | 46 | 55.42% | what causes ovarian cancer? |
| Dysplasia | 20 | 24.10% | what causes cervical dysplasia? |
| Risk Factors | 12 | 14.46% | how high are the chances of anal warts causing cancer of the cervix |
| Genetics | 4 | 4.82% | genes mitocondrial cause cancer cervix |
| Contraceptives | 1 | 1.20% | birth control pills and cervical cancer causes |

| **Symptoms** | Count | **Percent** | **Gynecological 5344 – Symptoms 939 17.57%** |
| --- | --- | --- | --- |
| Ovarian | 525 | 55.91% | symptons of overian cancer |
| Cervical | 243 | 25.88% | What are the symptoms of cervical cancer? |
| Uterine | 112 | 11.93% | what are the symptoms of uternine CANCER |
| Vaginal | 29 | 3.09% | Symptoms Of Vaginal Cancer |
| Endometrial | 26 | 2.77% | Endometrial Cancer Symptoms |
| Vulvar | 4 | 0.43% | symptoms of vulvar dysplasia |

| **Testing** | **Count** | **Percent** | **Gynecological 5344 – Testing 452 8.46%** |
| --- | --- | --- | --- |
| Biopsy | 228 | 50.44% | where can I find informatin on endometrial biopsies |
| Pap | 165 | 36.50% | do Pap smears detect ovarian cancer? |
| Unspecified | 27 | 5.97% | cervical cancer screening |
| MRI | 16 | 3.54% | Cervical Tumor Mri Pictures |
| Blood | 12 | 2.65% | Can cervical cancer be detected through blood tests |
| Sel-Diagnosis | 4 | 0.88% | vulva cancer self exam |

| **Treatment** | **Count** | **Percent** | **Gynecological 5344 – Treatment 247 4.62%** |
| --- | --- | --- | --- |
| Unspecified | 114 | 46.15% | treatment for ovarian cancer |
| Surgery | 21 | 8.50% | Ovarian Dermoid Laparoscopic surgery? |
| Chemotherapy | 19 | 7.69% | What is chemotherapy for cervical cancer? |
| Radiation | 18 | 7.29% | Where can i find information about radiation treatment for cervical cancer |
| Dysplasia | 17 | 6.88% | Treatment For Cervical Dysplasia |
| Hysterectomy | 16 | 6.48% | hystorectomy and cervical cancer |
| Medication | 14 | 5.67% | what brad name medications treat cervical cancer? |
| Alterantive | 12 | 4.86% | ALTERNATIVE OVARIAN CANCER TREATMENTS |
| LEEP | 8 | 3.24% | LEEP procedure for cervical cancer |
| Hormone | 4 | 1.62% | where can i find information about hormone cancer therapy for uterine cancer? |
| Stem Cell Transplant | 4 | 1.62% | ovarian cancer stem cell transplant |

**Head & Neck Cancers**

| **Head & Neck** | **Count** | **Percent** | **Head & Neck Total Count 2522** |
| --- | --- | --- | --- |
| General Information | 1485 | 58.88% | Where can I find information on cancers of the neck: |
| Pictures | 422 | 16.73% | Where can I find pictures of Throat Cancer on the web? |
| Symptoms | 418 | 16.57% | what are signs of mouth cancer? |
| Definition | 59 | 2.34% | what is throat cancer? |
| Treatment | 52 | 2.06% | alternative treatments for throat cancer |
| Cause/Risk/Link | 47 | 1.86% | Where can I find information on mouth cancer from chewing tobacco? |
| Diagnosis & Testing | 26 | 1.03% | where could i find role of CT Scan &MRI in testing for head & neck cancers |
| Statistics | 13 | 0.52% | What is the survival rate of throat cancer? |

| **General Information** | **Count** | **Percent** | **Head & Neck 2522 – General Info 1485 58.88%** |
| --- | --- | --- | --- |
| Throat | 512 | 34.48% | Where can I find information on throat cancer? |
| Mouth | 308 | 20.74% | Where can I find information on mouth cancer? |
| Oral | 307 | 20.67% | facts on oral cancers |
| Tongue | 188 | 12.66% | Base Of Tongue Cancer |
| Head | 113 | 7.61% | Please find me information on Head Cancer |
| Neck | 57 | 3.84% | Where can I find information on cancers of the neck: |

| **Symptoms** | **Count** | **Percent** | **Head & Neck 2522 – Symptoms 418 16.57%** |
| --- | --- | --- | --- |
| Throat | 267 | 63.72% | what are the signs and symptoms of throat cancer |
| Oral | 56 | 13.37% | What are the symptoms of oral cancer? |
| Mouth | 55 | 13.13% | what are the symptoms of mouth cancer |
| Tongue | 21 | 5.01% | what are the symptoms of tounge cancer |
| Neck | 16 | 3.82% | what are the symptoms of neck cancer? |
| Head | 4 | 0.95% | symptoms of cancer in the head region |

| **Definition** | **Count** | **Percent** | **Head & Neck 2522 – Definition 59 2.34%** |
| --- | --- | --- | --- |
| Throat | 31 | 52.54% | what is cancer of the throat? |
| Mouth | 16 | 27.12% | what is cancer of the mouth? |
| Oral | 8 | 13.56% | what is oral cancer? |
| Neck | 4 | 6.78% | what is cancer of the neck? |

**Hematologic & Blood Cancers**

| **Hematologic/Blood** | **Count** | **Percent** | **Hematologic & Blood Total Count 5448** |
| --- | --- | --- | --- |
| General Information | 3781 | 69.40% | can you give me any info on leukemia? |
| Definition | 706 | 12.96% | what is lukemia? |
| Symptoms | 539 | 9.89% | what are symptoms of leukemia |
| Treatments | 175 | 3.21% | What is the treatment for chronic leukemia? |
| Organizations | 102 | 1.87% | Multiple Myeloma Foundation |
| Cause/Risk/Link | 50 | 0.92% | Information linking leukemia to smoking |
| Diagnosis & Testing | 40 | 0.73% | how is leukemia diagnosed? |
| Statistics | 24 | 0.44% | Leukemia statistics |
| Prevention | 12 | 0.22% | how do you prevent leukemia? |
| Hospitals | 9 | 0.17% | hospitals for blood cancer over india |
| Coping | 6 | 0.11% | leukemia support group in San Diego CA |
| History | 4 | 0.07% | history of leukemia |

| **Treatment** | **Count** | **Percent** | **Hematologic & Blood 5448 – Treatment 175 3.21%** |
| --- | --- | --- | --- |
| Medications | 65 | 37.14% | what medication is used to treat leukemia |
| Unspecified Leukemia | 53 | 30.29% | treatment for leukemia |
| Unspecified Myeloma | 33 | 18.86% | Multiple Myeloma Treatment |
| Stem Cells | 10 | 5.71% | medicare guidelines for peripheral stem cell transplants for multiple myeloma |
| Alternative | 8 | 4.57% | what are some alternative treatments for leukemia |
| Umbilical Cells | 6 | 3.43% | LEUKEMIA UMBILICAL TREATMENT FOR ADULTS |

| **General Information** | **Count** | **Percent** | **Hematologic & Blood 5448 – General Info 3781 69.40%** |
| --- | --- | --- | --- |
| Leukemia | 2895 | 76.57% | I need information on lukemia |
| Myeloma | 592 | 15.66% | where can i get information on multiple myeloma |
| Bone Marrow | 148 | 3.91% | Bone Marrow Cancer |
| Blood Cancers | 146 | 3.86% | What disease is blood cancer? |

| **Symptoms** | **Count** | **Percent** | **Hematologic & Blood 5448 – Symptoms 539 9.89%** |
| --- | --- | --- | --- |
| Leukemia | 510 | 94.62% | what are symptoms of leukemia |
| Multiple Myeloma | 19 | 3.53% | what are some of the signs and symptoms of multiple myeloma |
| Blood | 5 | 0.93% | what are the symptoms of blood cancer |
| Amyloid Myeloma | 5 | 0.93% | what are the symptoms of amyloid myeloma |

**Lung Cancer**

| **Lung** | **Count** | **Percent** | **Lung Cancer Total Count 4630** |
| --- | --- | --- | --- |
| General Information | 3223 | 69.61% | where can i find information on lung cancer |
| Symptoms | 530 | 11.45% | What are the syptoms of lung cancer |
| Cause/Risk/Link | 305 | 6.59% | does smoking marijuana cause lung cancer |
| Treatment | 219 | 4.73% | what is the best treatment for lung cancer? |
| Definition | 150 | 3.24% | what is lung cancer |
| Statistics | 113 | 2.44% | how many people die of lung cancer every year? |
| Diagnosis & Testing | 90 | 1.94% | when does a doctor perform a lung biopsy? |

| **Cause/Risk/Link** | **Count** | **Percent** | **Lung Cancer 4630 – Cause/Risk/Link 305 6.59%** |
| --- | --- | --- | --- |
| Unspecified | 180 | 59.02% | what are the causes of lung cancer? |
| Smoking | 102 | 33.44% | does smoking cause lung cancer? |
| Marijuana | 23 | 7.54% | does marijuana cause lung cancer |

| **Diagnosis & Testing** | **Count** | **Percent** | **Lung Cancer 4630 – Diagnosis & Testing 90 1.94%** |
| --- | --- | --- | --- |
| Biopsy | 64 | 71.11% | when does a doctor perform a lung biopsy? |
| Unspecified | 18 | 20.00% | diagnosis of lung cancer |
| CAT/CT Scan | 8 | 8.89% | Ct Scan Of Lungs |

| **General Information** | **Count** | **Percent** | **Lung Cancer 4630 – General Info 3223 69.61%** |
| --- | --- | --- | --- |
| Unspecified | 2035 | 63.14% | where can I learn information regarding lung cancer |
| Pictures | 635 | 19.70% | Where cna I find pictures on lung cancer? |
| Types | 255 | 7.91% | What is the worst type of lung cancer to have? |
| Stages | 136 | 4.22% | what are the signs of final stages of lung cancer |
| Metastasize | 51 | 1.58% | what happens when lung cancer shows up in your bone marrow |
| Paper/Book | 40 | 1.24% | newspaper articles on lung cancer |
| History | 26 | 0.81% | what year was it that they linked smoking to lung cancer |
| Hospital/Clinic/Doctor | 18 | 0.56% | where can I find directory of lung cancer oncologists? |
| Websites | 18 | 0.56% | take me to some websites on lung cancer |
| Clinical Trials | 9 | 0.28% | Find Lung Cancer Trials |

| **Types** | **Count** | **Percent** | **Lung Cancer 4630 – Types 255 7.91%** |
| --- | --- | --- | --- |
| Small Cell | 102 | 40.00% | Where can I find information about small cell lung cancer? |
| Adenocarcinoma | 84 | 32.94% | WHAT IS ADENOCARCINOMA OF THE LUNGS |
| What are…. | 26 | 10.20% | what are the different types of lung cancers? |
| Squamous Cell | 24 | 9.41% | What is squamous cell lung carcinoma? |
| Non-Small Cell | 19 | 7.45% | NON SMALL CELL LUNG CANCER |

| **Statistics** | **Count** | **Percent** | **Lung Cancer 4630 – Statistics 113 2.44%** |
| --- | --- | --- | --- |
| Survival Rate | 45 | 39.82% | what is the survival rate for lung cancer |
| Unspecified | 22 | 19.47% | where can i find statistics on lung cancer? |
| UK | 13 | 11.50% | How many people in the uk get lung cancer a year |
| Smoking | 11 | 9.73% | What is the % chance you'll get lung cancer if you smoke |
| Life Expenctancy | 10 | 8.85% | lung cancer life expectency |
| Hong Kong | 8 | 7.08% | how many people die of lung cancer each year in Hong Kong? |
| France | 4 | 3.54% | lung cancer deaths in france |

| **Treatment** | **Count** | **Percent** | **Lung Cancer 4630 – Treatment 219 4.73%** |
| --- | --- | --- | --- |
| Unspecified | 118 | 53.88% | What are treatments for lung cancer? |
| Cure | 26 | 11.87% | is there a cure for lung cancer? |
| Medications | 21 | 9.59% | Drugs used for treating Lung Cancer |
| Radiation Therapy | 19 | 8.68% | Where in the US can a lung cancer patient recieve Proton Therapy |
| Alternative | 13 | 5.94% | korean alternative cures for lung cancer |
| Surgery | 10 | 4.57% | lung cancer surgery |
| Latest | 8 | 3.65% | what is the latest treatment for lung cancer? |
| Pallative | 4 | 1.83% | Pallative treatment for lung cancer patients? |

**Lymphomas**

| **Lymphomas** | **Count** | **Percent** | **Lymphomas Total Count 3333** |
| --- | --- | --- | --- |
| General Information | 2391 | 71.74% | Where do I go to find out about Lymphoma? |
| Definition | 580 | 17.40% | what is non-hodgkins lymphoma? |
| Symptoms | 187 | 5.61% | What are the symptoms of lymphoma? |
| Treatment | 55 | 1.65% | what are the best places for treatment of hodgkins lymphoma |
| Statistics | 36 | 1.08% | what are the chances for surviving hodgkins |
| Stages | 26 | 0.78% | Stages Non Hodgkins Lymphoma |
| Pictures | 20 | 0.60% | pictures of lymphoma |
| Websites | 11 | 0.33% | search for hodgkins diesease websites |
| Cause/Risk/Link | 10 | 0.30% | how do you get lymphoma |
| Celebrity | 8 | 0.24% | Famous people with hodgkins disease |
| Coping | 5 | 0.15% | non-hodgkins lymphoma support group |
| Diagnosis & Testing | 4 | 0.12% | what kind of scan is needed for non-hodkins lymphoma |

| **General Information** | **Count** | **Percent** | **Lymphomas 3333 – General Info 2391 71.74%** |
| --- | --- | --- | --- |
| Unspecified Lymphoma | 908 | 37.98% | where can i get information on lymphoma |
| Hodgkin's | 739 | 30.91% | where can I find information about Hodgkins lymphona |
| Non-Hodgkins | 421 | 17.61% | where can i find information on non-hodgkins lymphoma? |
| Type | 81 | 3.39% | what are the types of lymphoma |
| B Cell | 70 | 2.93% | i need info on high-grade b-cell lymphoma |
| T Cell | 58 | 2.43% | Human T-cell Lymphoma |
| Burkitt's | 51 | 2.13% | info about Burkitt's Lymphoma |
| Large Cell | 25 | 1.05% | where can I find info on large cell cleaved lymphoma? |
| MALT | 19 | 0.79% | can you find info on malt lymphoma |
| Mantle Cell | 10 | 0.42% | where can I find informatiojn on mantel cell lymphoma |
| Lymphoplasmacytoid | 9 | 0.38% | Lymphoplasmacytoid lymphoma |

| **Definition** |  |  |
| --- | --- | --- |
| Unspecified Lymphoma | 321 | 55.34% |
| Hodgkins | 147 | 25.34% |
| Non-Hodgkins | 57 | 9.83% |
| B Cell | 30 | 5.17% |
| T Cell | 17 | 2.93% |
| MALT | 8 | 1.38% |

| **Symptoms** | **Count** | **Percent** | **Lymphomas 3333 – Symptoms 187 5.61%** |
| --- | --- | --- | --- |
| Unspecified Lymphoma | 94 | 50.27% | what are the symptoms of lymphomia |
| Hodgkins | 87 | 46.52% | what are the symptoms of hodkins lymphoma |
| Non-Hodgkins | 6 | 3.21% | Are there any symtoms for non hodgkins lymphoma? |

**Miscellaneous Cancers**

| **Miscellaneous Cancers** | **Count** | **Percent** | **Miscellaneous Cancer Total Count 1633** |
| --- | --- | --- | --- |
| Endocrine | 901 | 55.17% | Where can I find information on papilary thyroid cancer? |
| Neoplasm | 272 | 16.66% | what is a neoplasm |
| Kaposis | 262 | 16.04% | Where can I find out about symptoms karposi sarcoma? |
| Ocular | 179 | 10.96% | what CAN YOU TELL ME ABOUT THE CANCER MELANOMA OF THE EYES? |
| Germ Cell | 19 | 1.16% | what is germ cell cancer? |

| **Endocrine** | **Count** | **Percent** | **Miscellaneous Cancer 1633 – Endocrine Specific 899 55.05%** |
| --- | --- | --- | --- |
| Thyroid | 511 | 56.84% | What are the symptoms of thyroid cancer? |
| Pheochromocytoma | 150 | 16.69% | WHAT IS PHEOCHROMOCYTOMA |
| Adrenals | 113 | 12.57% | Where can I find information on adrenal gland cancer? |
| Pituitary | 81 | 9.01% | where do I find an mri of a pituitary tumor? |
| General Information | 24 | 2.67% | where can I find info on Multiple Endocrine Neoplastia? |
| Islet Cell | 20 | 2.22% | what is islet cell carcinoma |

| **Endocrine Overall** | **Count** | **Percent** | **Miscellaneous Cancer 1633 – Endocrine 901 55.17%** |
| --- | --- | --- | --- |
| General Information | 679 | 75.36% | Where can I find information on papilary thyroid cancer? |
| Symptoms | 88 | 9.77% | What are the symtoms of thyroid cancer ? |
| Definition | 64 | 7.10% | what is endocrine cancer? |
| Diagnosis & Testing | 41 | 4.55% | How do they do a thyroid biopsy? |
| Treatment | 16 | 1.78% | what is the latest treatment of adrenacortical carcinoma? |
| Benign | 5 | 0.55% | Benign Pituitary Gland Tumors |
| Metastatic | 4 | 0.44% | what is metastatic adrenocarcinoma? |
| Pictures | 4 | 0.44% | Where can i get pictures of thyroid cancer |

| **Kaposis** | **Count** | **Percent** | **Miscellaneous Cancer 1633 – Kaposi’s 262 16.04%** |
| --- | --- | --- | --- |
| General Information | 141 | 53.82% | examples of karposi's sarcoma |
| Pictures | 79 | 30.15% | where can I find a picture of kaposi's sarcoma? |
| Definition | 24 | 9.16% | what is kaposis sacroma |
| Symptoms | 18 | 6.87% | Where can I find out about symptoms karposi sarcoma? |

| **Neoplasm** | **Count** | **Percent** | **Miscellaneous Cancer 1633 – Neoplasm 272 16.66%** |
| --- | --- | --- | --- |
| General Information | 136 | 50.00% | Where can I find information on Neoplasam? |
| Definition | 112 | 41.18% | what does neoplasm mean |
| Malignant | 14 | 5.15% | information on malignant neoplasms |
| Metastatic | 6 | 2.21% | metastatic neoplasms |
| Benign | 4 | 1.47% | what is a benign neoplasm? |

| **Ocular** | **Count** | **Percent** | **Miscellaneous Cancer 1633 – Ocular 179 10.96%** |
| --- | --- | --- | --- |
| Unspecified Information | 56 | 31.28% | Where can I find more information on eye cancer in general? |
| Melanoma | 52 | 29.05% | what is melanoma of the eye |
| Tumors | 23 | 12.85% | info about eye tumors |
| Retinoblastoma | 20 | 11.17% | What is retinoblastoma? |
| Glioma | 16 | 8.94% | Optic Nerve Glioma |
| Optic Dysplasia | 12 | 6.70% | what is septo optic dysplasia? |

**Pediatric Cancers**

| **Pediatrics** | **Count** | **Percent** | **Pediatric Total Count 603** |
| --- | --- | --- | --- |
| Type | 403 | 66.83% | Where can I find out about childhood leukemia? |
| General Information | 81 | 13.43% | where can i find information on children's cancers? |
| Coping | 51 | 8.46% | Summer camps in Maine for children with cancer |
| Organization | 25 | 4.15% | how much money did National childhood cancer foundation raise last yaer? |
| Donate Hair | 16 | 2.65% | where can I donate my hair to make wigs for children that go throught chemotherpy? |
| Statistics | 12 | 1.99% | how many children die of cancer each year? |
| Treatment | 8 | 1.33% | cancer treatments for children |
| Diagnosis & Testing | 7 | 1.16% | What are the first signs and symptoms of childhood cancer? |

| **Coping** | **Count** | **Percent** | **Pediatric 603 – Coping 51 8.46%** |
| --- | --- | --- | --- |
| Support Groups | 36 | 70.59% | where can I find parent support groups for kids with leukemia |
| Family | 10 | 19.61% | how to explain to a child they have cancer |
| Camps | 5 | 9.80% | Summer camps in Maine for children with cancer |

| **Cancer Types** | **Count** | **Percent** | **Pediatric 603 – 403 66.83%** |
| --- | --- | --- | --- |
| Hematologic/Blood | 137 | 34.00% | Where can I find out about childhood leukemia? |
| Neuroblastoma | 133 | 33.00% | where can i find information on the cancer neuroblastoma? |
| Rhabdomyosarcoma | 68 | 16.87% | what is rhabdomyosarcoma? |
| Wilms | 18 | 4.47% | what is wilm's tumor |
| Skin | 13 | 3.23% | what are the effects of skin cancer on young children |
| Digestive/Gastro/Bowel | 10 | 2.48% | can infants get liver cancer |
| Bone | 9 | 2.23% | Where can I find information about bone cancer in children? |
| Gynecological | 6 | 1.49% | can Cervical Cancer be passed on to children |
| Hodkins Lymphoma | 5 | 1.24% | childhood hodgkins disease |
| Brain/Neurological | 4 | 0.99% | brain neoplasms AND children |

**Skin Cancers**

| **Skin Cancer** | **Count** | **Percent** | **Skin Cancer Total Count 6709** |
| --- | --- | --- | --- |
| General Information | 3596 | 53.60% | Where can I find information regarding skin cancers? |
| Cancer Type | 2157 | 32.15% | i need information on melanoma. |
| Symptoms | 298 | 4.44% | what are symptoms of skin cancer |
| Cause/Risk/Link | 169 | 2.52% | Is there a type of skin cancer caused by grease from cars? |
| Treatments | 161 | 2.40% | what are some skin cancer treatments |
| Diagnosis & Testing | 132 | 1.97% | Diagnosis of Skin cancer |
| Statistics | 105 | 1.57% | What was the number of skin cancer cases in 1992 |
| Prevention | 60 | 0.89% | How To Prevent Skin Cancer |
| Organization | 16 | 0.24% | skin cancer foundations |
| Coping | 15 | 0.22% | where can i find informatation on pain management of skin cancer |

| **General Information** | **Count** | **Percent** | **Skin Cancer 6709 – General Information 3596 53.60%** |
| --- | --- | --- | --- |
| Unspecified | 2554 | 71.02% | where can i findx info on skin cancer |
| Pictures | 981 | 27.28% | where can i find photos of skin cancer |
| History | 35 | 0.97% | History Of Skin Cancer |
| Websites | 15 | 0.42% | web sites on skin cancer |
| Clinical Trials | 11 | 0.31% | clinical trials on skin cancers |

| **Cancer Type** | **Count** | **Percent** | **Skin Cancer 6709 – Cancer Type 2157 32.15%** |
| --- | --- | --- | --- |
| Melanomas | 1707 | 79.14% | Where can I find information about Melanoma? |
| Basal Cell | 322 | 14.93% | Where can I find information on "basal cell carcinoma?" |
| Inquiry | 128 | 5.93% | what are the types of skin cancer |

| **Cause/Risk/Link** | **Count** | **Percent** | **Skin Cancer 6709 – Cause/Risk/Link 169 2.52%** |
| --- | --- | --- | --- |
| Sun/Tanning Beds | 60 | 35.50% | Can You get skin cancer from sunbeds? |
| Unspecified | 41 | 24.26% | What causes skin cancer? |
| Ozone Hole | 31 | 18.34% | Does the depletion of the ozone layer cause the increase in skin cancers? |
| Moles | 29 | 17.16% | moles and skin cancer? |
| Grease/Oil | 8 | 4.73% | Does grease cause skin cancer? |

| **Diagnosis & Testing** | **Count** | **Percent** | **Skin Cancer 6709 – Diagnosis & Testing 132 1.97%** |
| --- | --- | --- | --- |
| Unspecified | 69 | 52.27% | Diagnosis of Skin cancer |
| Biopsy | 30 | 22.73% | Where can I find imformation on skin biopsy? |
| Self-Diagnosis | 28 | 21.21% | Where can I learn about the skin cancer self-examination? |
| Melanoma | 5 | 3.79% | Malignant Melanoma Diagnosis |

**Soft Tissue Cancers**

| **Soft Tissue** | **Count** | **Percent** | **So****ft Tissue Total Count 3954** |
| --- | --- | --- | --- |
| Dysplasia | 1122 | 28.38% | where can i find information on mild dysplasia? |
| Sarcoma | 717 | 18.13% | indormation on soft tissue sarcoma |
| Tumor | 657 | 16.62% | Where can I find information on cancer tumors under toe nails? |
| Ganglion | 624 | 15.78% | what about a ganglion cyst of the toe? |
| Dermoid | 432 | 10.93% | Where can I find information on dermoid cysts? |
| Fibroid | 277 | 7.01% | Where can I find information on Fibroid tumors? |
| Oligodendroglioma | 80 | 2.02% | find me information about Oligodendroglioma Tumors |
| Soft Tissue | 25 | 0.63% | articles on fatty ssoft tissue tumors |
| Glioma | 20 | 0.51% | please find information on gliomas cancer |

| **Dermoid** | **Count** | **Percent** | **Soft Tissue 3954 – Dermoid 432 10.93%** |
| --- | --- | --- | --- |
| General Information | 329 | 76.16% | Where can I find information on dermoid cysts? |
| Definition | 60 | 13.89% | what is a dermoid cyst? |
| Cause/Risk/Link | 29 | 6.71% | Information Regarding Dermoid Cyst Cause |
| Treatment | 14 | 3.24% | How do I prepare for dermoid removal surgery? |

| **Dysplasia** | **Count** | **Percent** | **Soft Tissue 3954 – Dysplasia 1122 28.38%** |
| --- | --- | --- | --- |
| General Information | 637 | 56.77% | where can i find information on dysplasia |
| Definition | 192 | 17.11% | what is dysplasia |
| Anatomy | 161 | 14.35% | where can I find information on hip dysplasia? |
| Myelodysplasia | 52 | 4.63% | can you tell me about myelo dysplasia? |
| Mild/Severe | 40 | 3.57% | where can i find information on mild dysplasia? |
| Treatment | 28 | 2.50% | information on cryosurgery for dysplasia |
| Cause/Risk/Link | 8 | 0.71% | how do you get dysplasia |
| Symptoms | 4 | 0.36% | what are some signs of dysplasia |

| **Fibroid** | **Count** | **Percent** | **Soft Tissue 3954 – Fibroid 277 7.01%** |
| --- | --- | --- | --- |
| General Information | 215 | 77.62% | Where can I find information on Fibroid tumors? |
| Definition | 25 | 9.03% | what is fibroid cancer |
| Symptoms | 16 | 5.78% | what are the symptoms of fibroid tumors |
| Treatment | 11 | 3.97% | Wholistic Treatment of Fibroid Tumors |
| Diagnosis & Testing | 10 | 3.61% | how do you diagnose fibroid cancer |

| **Ganglion** | **Count** | **Percent** | **Soft Tissue 3954 – Ganglion 624 15.78%** |
| --- | --- | --- | --- |
| General Information | 353 | 0.565705 | Where can I find information on a ganglion? |
| Definition | 126 | 0.201923 | what are ganglion cancers? |
| Anatomy | 117 | 0.1875 | what about a ganglion cyst of the toe? |
| Treatment | 28 | 0.044872 | Type of Ganglion Cysts Treatments |

| **Sarcoma** | **Count** | **Percent** | **Soft Tissue 3954 – Sarcoma 717 18.13%** |
| --- | --- | --- | --- |
| General Information | 434 | 60.53% | indormation on soft tissue sarcoma |
| Definition | 77 | 10.74% | what is a sarcoma? |
| Leiomyo- | 76 | 10.60% | what is leiomyosarcoma |
| Fibro- | 44 | 6.14% | tell me about fibrosarcoma |
| Angio- | 41 | 5.72% | what is angiosarcoma |
| Treatment | 27 | 3.77% | What herbs can be used to shrink canceroussarcomas>? |
| Statistics | 10 | 1.39% | info on nebraska sarcoma Mortality Rate |
| Symptoms | 8 | 1.12% | What are symptoms of sarcomas? |

| **Tumor** | **Count** | **Percent** | **Soft Tissue 3954 – Tumor 657 16.62%** |
| --- | --- | --- | --- |
| General Information | 233 | 35.46% | Where can I find information on Cancer Tumors? |
| Definition | 93 | 14.16% | what is a tumor |
| Fatty | 64 | 9.74% | I need information on fatty tumors |
| Anatomy | 57 | 8.68% | What kind of tumors can be in the tendons of a hand or foot? |
| Pseudo | 54 | 8.22% | where can i find information about a pseudo tumor |
| Phyllodes | 36 | 5.48% | research on Sarcoma Phylloides Tumor |
| Lipoma | 32 | 4.87% | Where can I find information about Lipoma Tumor |
| Benign | 31 | 4.72% | articles on benign tumor |
| Pictures | 11 | 1.67% | WHAT DOES A TUMOR LOOK LIKE |
| Mast | 11 | 1.67% | mast cell tumors |
| Markers | 8 | 1.22% | what are tumor markers |
| Sheath | 8 | 1.22% | nerve sheath tumor |
| Malignant | 7 | 1.07% | research regarding malignant tumors |
| Non-Malignant | 6 | 0.91% | Non Malignant Tumors Resaerch |
| Wharton | 6 | 0.91% | where can i get info on a whartin tumor cancer |

**Treatment**

| **Treatment Overall** | **Count** | **Percent** | **Treatment Total Count 3832** |
| --- | --- | --- | --- |
| Treatment Type | 3223 | 84.11% | Is taichi used as an alternative treatment for cancer |
| General Information | 429 | 11.20% | where can i find information on cancer treatment? |
| Centers | 97 | 2.53% | cancer treatment centers of america |
| Latest | 22 | 0.57% | new treatments for cancer |
| Secondary | 20 | 0.52% | Restoring Taste Loss From Cancer Treatment |
| Side Effects | 20 | 0.52% | where can I find the side effects of treatments of cancer? |
| Local | 12 | 0.31% | cancer treatments in canada |
| Costs | 9 | 0.23% | what is the average treatment cost for cancer? |

| **Treatment Type** | **Count** | **Percent** | **Treatment 3832 – Treatment Type 3223 84.11%** |
| --- | --- | --- | --- |
| Chemotherapy | 2275 | 70.59% | what is chemotherapy oral form |
| Radiation Therapy | 534 | 16.57% | radiology implants for cancer treatement |
| Alternative | 239 | 7.42% | Is taichi used as an alternative treatment for cancer |
| Surgery | 127 | 3.94% | cancer amputation surgery |
| Steroids | 16 | 0.50% | steroids in cancer treatment |
| Experimental | 8 | 0.25% | experimental cancer treatments |
| Genetics | 8 | 0.25% | genetics in the treatment of cancer |
| Gamma Knife | 7 | 0.22% | gamma knife for cancer treatment |
| Laser | 5 | 0.16% | laser in treating cancer |
| Protons | 4 | 0.12% | where can I find information on cancer treatment with protons? |

| **Alternative** | **Count** | **Percent** | **Treatment 3832 – Treatment Type 3223 84.11% - Alternative 239 7.42%** |
| --- | --- | --- | --- |
| "Alternative" | 121 | 50.63% | Alternative Cancer Treatment |
| Homeopathy | 66 | 27.62% | homeopathic treatments for cancer |
| Herbal | 13 | 5.44% | herbs for cancer treatment |
| Nutrition | 10 | 4.18% | High protein foods for cancer treatment |
| Massage | 7 | 2.93% | can massage help people with cancer |
| Taichi | 6 | 2.51% | Is taichi used as an alternative treatment for cancer |
| Enzyme | 6 | 2.51% | enzyme cancer treatment |
| Vitamins | 5 | 2.09% | what vitamins are indicated in the treatment of cancer |
| Decompression | 5 | 2.09% | decompression in cancer treatment |

| **Chemotherapy** | **Count** | **Percent** | **Treatment 3832 – Treatment Type 3223 84.11% - Chemotherapy 2275 70.59%** |
| --- | --- | --- | --- |
| Unspecified | 951 | 41.82% | where can I found information about cancer drugs? |
| Methotrexate | 312 | 13.72% | I need information on the drug methotrexate? |
| Interferon | 257 | 11.30% | What diseases is Interferon Therapy used to treat? |
| Side Effects | 232 | 10.20% | what are the side effects of intense chemotherapy |
| Definition | 162 | 7.12% | what is chemothereaphy? |
| Taxol | 136 | 5.98% | where can I find infomation on the chemistry of Taxol? |
| New/Experimental | 111 | 4.88% | what are the newest cancer drugs |
| Pictures | 26 | 1.14% | where can i find pictures relating to chemotheraphy |
| Cost | 18 | 0.79% | How much does chemotherapy cost? |
| Taxotere | 18 | 0.79% | where can I find ifo on taxtere chemotherapy |
| Drug List | 16 | 0.70% | list chemotherapeutic agents |
| History | 12 | 0.53% | Where can I find how chemotherapy began |
| Carboplatin | 5 | 0.22% | carboplatin chemotherapy treatment information online |
| Cobalt | 4 | 0.18% | Cobalt treatment for cancer |
| Oral | 4 | 0.18% | what is chemotherapy oral form |
| Rituximab | 3 | 0.13% | Cancer Drug Rituximab Research Online |
| Aromasin | 3 | 0.13% | where can I find information on the cancer drug Aromagin? |
| Cisplatin | 2 | 0.09% | where can i find information about cisplantin the chemotherapy drug |
| Naltrexone | 2 | 0.09% | information on naltrexone to treat cancer |
